# Mucosal fluid glycoprotein DMBT1 suppresses twitching motility and virulence of the opportunistic pathogen *Pseudomonas aeruginosa*

**DOI:** 10.1371/journal.ppat.1006392

**Published:** 2017-05-10

**Authors:** Jianfang Li, Matteo M. E. Metruccio, David J. Evans, Suzanne M. J. Fleiszig

**Affiliations:** 1 School of Optometry, University of California, Berkeley, California, United States of America; 2 College of Pharmacy, Touro University California, Vallejo, California, United States of America; 3 Graduate Groups in Vision Science, Microbiology and Infectious Disease & Immunity, University of California, Berkeley, California, United States of America; The University of Texas at Austin, UNITED STATES

## Abstract

It is generally thought that mucosal fluids protect underlying epithelial surfaces against opportunistic infection via their antimicrobial activity. However, our published data show that human tear fluid can protect against the major opportunistic pathogen *Pseudomonas aeruginosa* independently of bacteriostatic activity. Here, we explored the mechanisms for tear protection, focusing on impacts of tear fluid on bacterial virulence factor expression. Results showed that tear fluid suppressed twitching motility, a type of surface-associated movement conferred by pili. Previously, we showed that twitching is critical for *P*. *aeruginosa* traversal of corneal epithelia, exit from epithelial cells after internalization, and corneal virulence. Inhibition of twitching by tear fluid was dose-dependent with dilutions to 6.25% retaining activity. Purified lactoferrin, lysozyme, and contrived tears containing these, and many other, tear components lacked the activity. Systematic protein fractionation, mass spectrometry, and immunoprecipitation identified the glycoprotein DMBT1 (Deleted in Malignant Brain Tumors 1) in tear fluid as required. DMBT1 purified from human saliva also inhibited twitching, as well as *P*. *aeruginosa* traversal of human corneal epithelial cells *in vitro*, and reduced disease pathology in a murine model of corneal infection. DMBT1 did not affect PilA expression, nor bacterial intracellular cyclicAMP levels, and suppressed twitching motility of *P*. *aeruginosa* chemotaxis mutants (*chpB*, *pilK*), and an adenylate cyclase mutant (*cyaB*). However, dot-immunoblot assays showed purified DMBT1 binding of pili extracted from PAO1 suggesting that twitching inhibition may involve a direct interaction with pili. The latter could affect extension or retraction of pili, their interactions with biotic or abiotic surfaces, or cause their aggregation. Together, the data suggest that DMBT1 inhibition of twitching motility contributes to the mechanisms by which mucosal fluids protect against *P*. *aeruginosa* infection. This study also advances our understanding of how mucosal fluids protect against infection, and suggests directions for novel biocompatible strategies to protect our surface epithelia against a major opportunistic pathogen.

## Author summary

*Pseudomonas aeruginosa* is an opportunistic pathogen that causes life-threatening infections. *P*. *aeruginosa* disease is increasing in prevalence while bacteria continue to evolve antibiotic resistance. It is not clear how mucosal fluids usually protect against opportunistic pathogens. Knowing the key ingredients would help us understand susceptibility and develop novel biocompatible therapeutics. Mucosal fluid factors suppressing bacterial virulence may induce less bacterial resistance than traditional antimicrobials. Here we show that DMBT1, an abundant mucosal fluid glycoprotein, enabled tear fluid to inhibit *P*. *aeruginosa* twitching motility. We also show DMBT1 directly binds pili, which mediate twitching motility, suggesting a potential mechanism for twitching inhibition. Reflecting the known importance of twitching motility in virulence, purified DMBT1 reduced *P*. *aeruginosa* traversal of human cornea epithelial cell layers *in vitro*, and protected against *P*. *aeruginosa* induced disease *in vivo*, as does whole human tear fluid. These findings contribute to our understanding of mucosal fluid protection against infection, and suggest that DMBT1, or its derivatives, have potential as novel anti-virulence agents that protect against infection.

## Introduction

*Pseudomonas aeruginosa* is a Gram-negative opportunistic pathogen ubiquitous in our environment. It is a leading cause of life-threatening infections in debilitated individuals in the hospital setting [1], and of sight-threatening corneal disease in healthy people who wear contact lenses [2, 3]. However, the mechanism(s) by which medical devices at any mucosal surface predispose to infection with *P*. *aeruginosa* or other opportunists remains poorly understood [4, 5].

The surface of the eye is normally bathed in tear fluid, which like other mucosal fluids contains many proteins-peptides, lipids, small molecule metabolites, and electrolytes. Indeed, more than 1000 proteins have been identified in healthy human tear fluid [6]. In addition to playing antimicrobial roles, mucosal fluids function to provide lubrication, remove foreign debris, provide homeostatic factors, and repair epithelial damage [7], Our previous studies have confirmed that tear fluid collected from healthy people can protect corneas (of mice) against *P*. *aeruginosa* infection *in vivo* [8].

Of likely relevance to the pathogenesis of contact lens related infections, when a contact lens is worn it dramatically reduces normal tear exchange between the greater tear fluid reservoir and the space between the lens and ocular surface [9, 10]. Suggesting that tear fluid biochemistry is altered under a worn lens, and that this is potentially relevant to the pathogenesis of infection, bacteria inoculated on the back surface of worn lenses grew more efficiently after 8 h of wear compared to 1 h of wear [11]. Candidate antimicrobial tear components in tear fluid that could be impacted by lens wear include; complement, defensins, lactoferrin, lipocalin, lysozyme, secretory phospholipase A2, secretory IgA, soluble mucins (Muc5AC), and/or surfactant proteins (SP-A, SP-D) [12].

However, mechanisms other than antimicrobial activity can also contribute to the protective activity of tear fluid against *P*. *aeruginosa* virulence. Indeed, only ~ 50% of *P*. *aeruginosa* clinical isolates are susceptible to tear fluid bacteriostatic activity [13], but almost all show reduced virulence in tear fluid. Further, these tear fluid activities are mechanistically separable [13]. Relevant to this, tear fluid can act directly on epithelial cells to enhance their resistance to *P*. *aeruginosa* virulence [13, 14] through upregulation of epithelial cell innate defense factors, e.g. RNase7 and ST2 [14, 15], alterations to microRNA expression [14], and the regulation of transcription factors NFκB and AP-1 [14]. Non-bacteriostatic activities of tear fluid also include its capacity to disperse *P*. *aeruginosa* biofilms [16], which are thought to be key to the pathogenesis of device-related infections.

A multitude of *P*. *aeruginosa* virulence factors can participate in virulence during corneal infection, most playing redundant roles but some required for full virulence [4, 17]. Among the major contributions is twitching motility [18, 19]. Twitching motility is a surface-associated bacterial movement conferred by extension and retraction of type IV pili (T4P) commonly used by Gram-negative bacteria [20]. While assisting bacterial adhesion to surfaces, retraction of pili can bring bacteria into intimate contact with the surface, allowing it to migrate away from the initial point of contact or toward an attractant, to reposition cells with respect to one another (e.g. differentiation within a biofilm), and can also help cells efficiently escape from surfaces when desirable [21]. In *P*. *aeruginosa*, the T4P consist of a polymer of the PilA major pilin subunit, while the extension and retraction of pili are controlled by ATPases PilB, PilU and PilT [22, 23]. While twitching motility mutants are able to adhere to, and invade, human corneal epithelial cells grown *in vitro*, they have a reduced capacity to exit cells after invasion [19]. Twitching mutants are also defective in their ability to traverse multilayers of epithelial cells [19], which may explain their lack of virulence *in vivo* [18].

Given the protective effect of tear fluid against *P*. *aeruginosa*, and the critical role of twitching in corneal pathogenesis, we examined the impact of tear fluid on twitching motility. Systematic fractionation of human tear fluid, combined with mass spectrometry and immuno-precipitation, identified DMBT1 (also known as glycoprotein-340) as required for tear inhibition of twitching motility. DMBT1 purified from saliva was sufficient when used alone for inhibiting twitching, preventing *P*. *aeruginosa* traversal of multilayered epithelial *in vitro*, and for reducing corneal disease severity in a murine model of *P*. *aeruginosa* keratitis. These results suggest a novel function for mucosal fluid and specifically DMBT1 in innate defense against infection.

## Results

### Human tear fluid inhibits *P*. *aeruginosa* twitching motility

**Fig 1:**
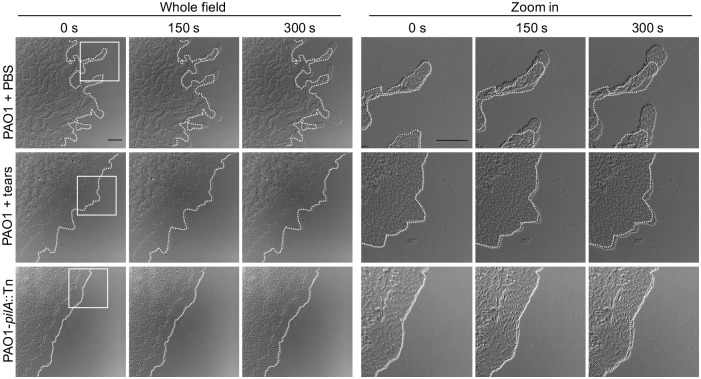
Human tear fluid inhibition of *P*. *aeruginosa* twitching motility. Captured frames of *P*. *aeruginosa* PAO1 twitching motility in a 5 min video after 4 h incubation on twitching media absorbed with PBS or undiluted human tear fluid. The twitching mutant PAO1-*pilA*::Tn served as a negative control. A 5 min time-lapse video was captured at 10 s intervals using a 60 × oil-immersion lens. Dotted lines indicate initial colony edges. Scale bar = 20 μm.

Time-lapse imaging was used to examine the impact of human tear fluid on twitching motility of *P*. *aeruginosa* strain PAO1. For this purpose, specialized agar media was used, absorbed with undiluted human tear fluid or PBS (see Methods). For each sample, images were collected with the colony edge positioned half way across the field. To determine speed of bacterial movement, time-lapse imaging was done by repeated 10 second interval image capture over a period of 300 seconds. Velocity of the twitching competent *P*. *aeruginosa* strain PAO1 was compared with and without tear fluid added to the media. PAO1-*pilA*::Tn, lacking twitching motility, was used as a negative control. Fig 1 shows the edge of representative bacterial colonies over a period of 300 seconds (0, 150 and 300 seconds) for PAO1 with and without tear fluid, and compared to the *pilA* twitching mutant control. Comparison of the colony edge at each time point relative to the dotted white line illustrating the colony edge at the start of the experiment, showed that tear fluid significantly reduced twitching motility compared to PBS (Fig 1, S1, S2 and S3 Videos).

**Fig 2:**
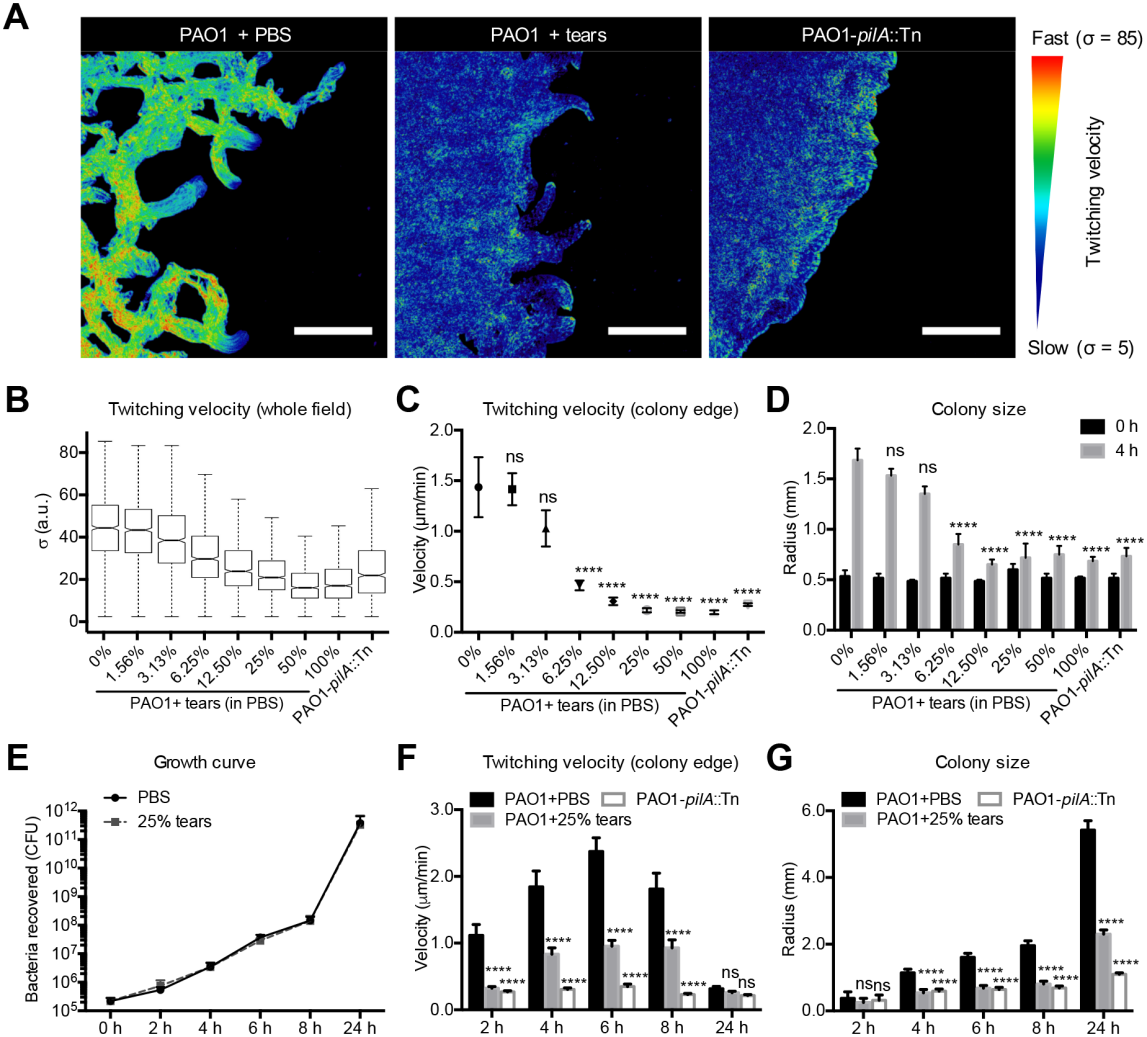
Tear fluid inhibition of *P*. *aeruginosa* twitching motility in a dose-dependent manner. **(A)** PAO1 motility was measured as the standard deviation (σ) of pixel intensity in a 5 min video. Larger standard deviations correspond to greater pixel intensity modulation resulting from higher bacterial motility. Blue represents slower movement. Scale bar = 50 μm. **(B)** Effect of tear fluid concentration on PAO1 twitching motility quantified using the above method. **(C)** Effect of tear fluid concentration on PAO1 twitching quantified by displacement of bacteria in the leading edge of the colony from the first to last slide of the same experiment shown in panel B. Ten single bacteria in the leading edge in each video were tracked. **(D)** Effect of tear fluid concentration on PAO1 colony size at time zero and 4 h incubation times on twitching media in the same experiment shown in panel B. **(E)** *P*. *aeruginosa* PAO1 growth on twitching media in PBS or 25% tear fluid. **(F)** PAO1 twitching velocity measured in 5 min videos on twitching media with PBS or 25% human tear fluid after different incubation times. **(G)** PAO1 colony size expansion on twitching media with PBS or 25% human tear fluid after different incubation times. In each panel, data are expressed as the mean ± SEM per sample from at least three independent experiments. Significance was determined by one-way ANOVA with Tukey's post-hoc analysis for twitching velocity and growth, and two-way ANOVA with Tukey's post-hoc analysis for colony size. ****, *P* < 0.0001; ***, *P* < 0.001; **, *P* < 0.01; *, *P* < 0.05; ns, not significant.

Fig 2 shows quantitative analysis of the impact of tear fluid on reducing twitching motility quantitatively using three methods; i) averaging velocity of all bacteria in the field i.e., bacterial motility in the whole field was corresponded to the standard deviation (σ) of pixel intensity in a 5 min movie (Fig 2A and 2B), ii) examining movement of 10 individual bacteria at the colony edge in a 5 min movie to better represent the concerted movements of twitching [24] (Fig 2C), and iii) quantifying colony size over time for 3 colonies per sample, based on published data that surface-associated twitching movement promotes colony expansion on solid surfaces [21] (Fig 2D). In each instance, tear fluid exposure caused a dose-dependent inhibition of twitching motility (as measured by reduced bacterial velocity and colony size expansion) after 4 h. Tear fluid dilutions up to and including as low as 6.25% retained some degree of inhibition (Fig 2C and 2D). Controls confirmed that tear fluid at 25% dilution, which inhibited twitching motility similarly to undiluted tears, did not inhibit bacterial growth (Fig 2E).

Since we had previously shown that *P*. *aeruginosa* could overcome the cytoprotective activities of tear fluid with prolonged exposure (8 h or more) [13], we also examined the impact of tear fluid (25%) on PAO1 twitching motility for up to 24 h. Tear fluid (25%) maintained inhibition of twitching for up to 8 h (Fig 2F and 2G). At 24 h, twitching velocity was very slow in PBS, and not significantly different from tear fluid-treated PAO1 or the *pilA* mutant (Fig 2F). However, tear fluid retained significant inhibition of colony size expansion at 24 h (Fig 2G). While that result likely reflected the cumulative effects of tear-mediated twitching inhibition, it suggested that the bacteria do not readily adapt to tear inhibition of twitching, or that the tear factor(s) involved are not readily compromised.

**Fig 3:**
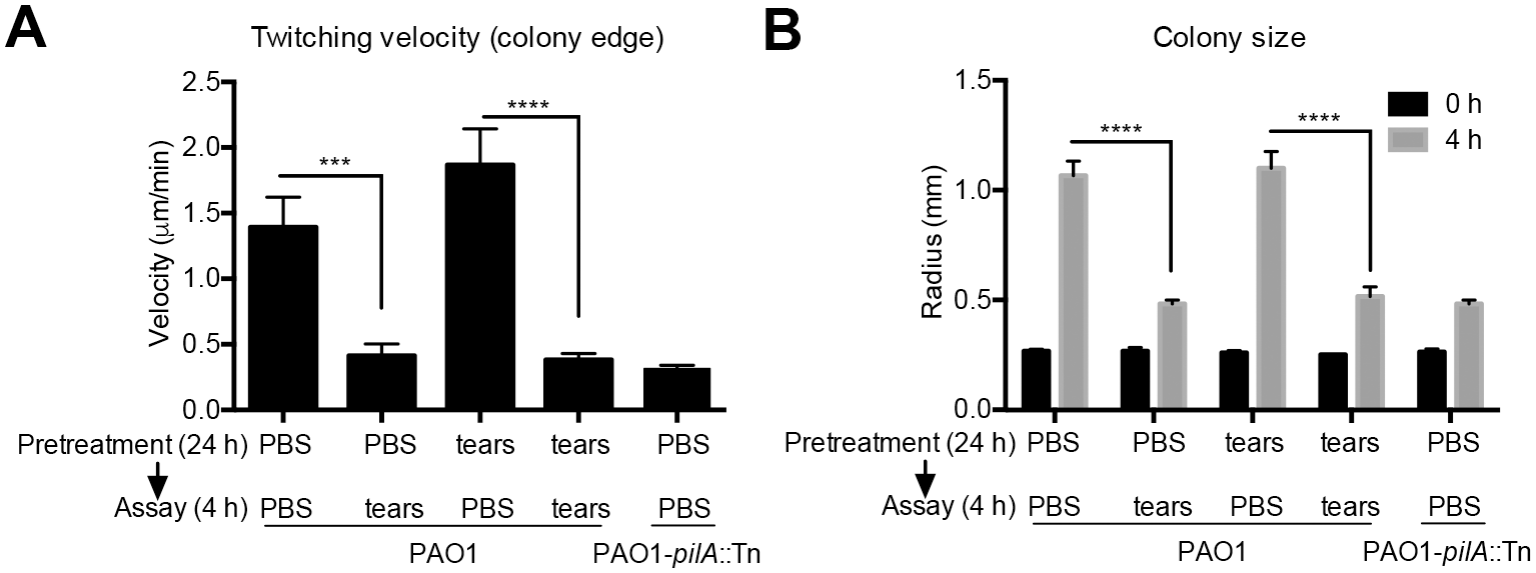
*P*. *aeruginosa* does not adapt to tear fluid inhibition of twitching motility. PAO1 was pretreated with PBS or 25% human tear fluid for 24 h on twitching media, then transferred to new twitching media with PBS or 25% human tear fluid for 4 h. Bacterial twitching velocity in 5 min videos (A) and colony size expansion (B) were measured. Data are shown as the mean ± SEM per sample from three independent experiments. Significance was determined using two-way ANOVA with Tukey's post-hoc analysis. ****, *P* < 0.0001; ***, *P* < 0.001; ns, not significant.

The above hypotheses were supported by experiments in which *P*. *aeruginosa* was pretreated with 25% tear fluid on twitching media for 24 h, before transfer to fresh media (PBS or 25% tear fluid) for 4 h. Results showed that 24 h tear pretreatment did not affect bacterial susceptibility to inhibition of twitching as measured by twitching velocity (Fig 3A) or colony size expansion (Fig 3B). The data also showed that tear-exposed *P*. *aeruginosa* recovered twitching once placed in PBS indicating reversible inhibition.

**Fig 4:**
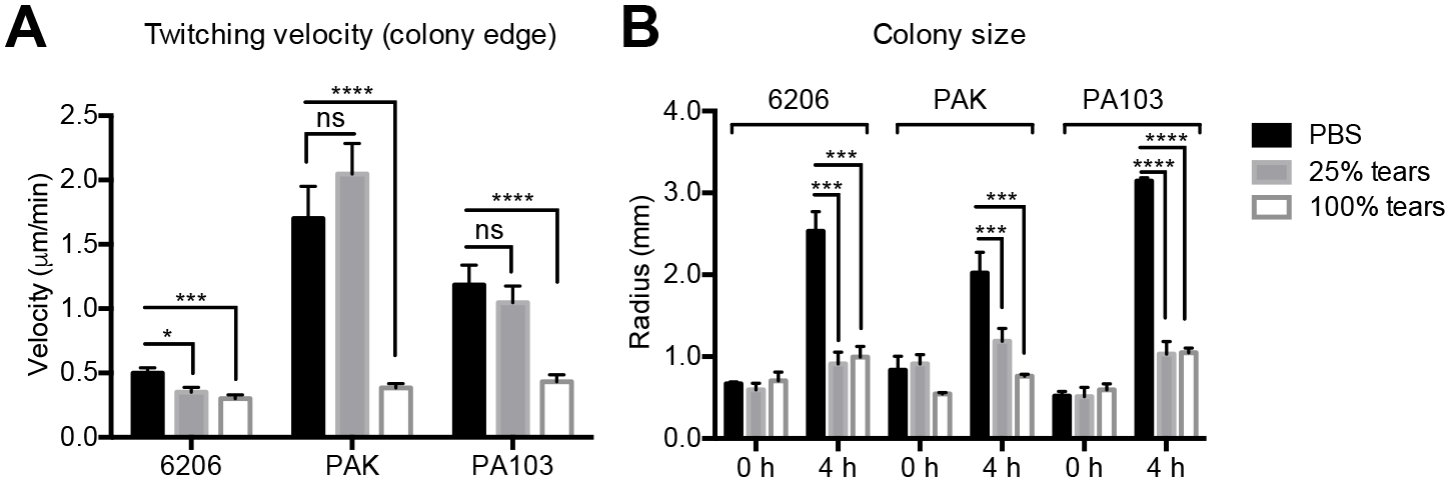
Human tear fluid inhibition of twitching motility on multiple *P*. *aeruginosa* strains. **(A)** Twitching velocity of *P*. *aeruginosa* strains 6206, PAK, and PA103 under the same experimental conditions used for PAO1. **(B)** Colony size on twitching media for *P*. *aeruginosa* strains 6206, PAK, and PA103 at 0 and 4 h incubation times. In each panel, data are expressed as the mean ± SEM per sample from at least three independent experiments. Significance was determined by one-way ANOVA with Tukey's post-hoc analysis for twitching velocity and growth, and two-way ANOVA with Tukey's post-hoc analysis for colony size. ****, *P* < 0.0001; ***, *P* < 0.001; *, *P* < 0.05; ns, not significant.

Several other *P*. *aeruginosa* strains were also tested for susceptibility to tear inhibition of twitching motility (Fig 4). Strains 6206, PAK, and PA103 were each susceptible to tear inhibition as measured by reduced velocity (Fig 4A) or reduced colony size expansion (Fig 4B). Some variability was noted between these strains and PAO1. For example, undiluted tear fluid was required for reducing twitching velocity of strains PAK and PA103. Nevertheless, these data show that tear fluid inhibition of twitching motility is not restricted to one strain.

### Identification of DMBT1 as a tear fluid factor inhibiting twitching motility

**Fig 5:**
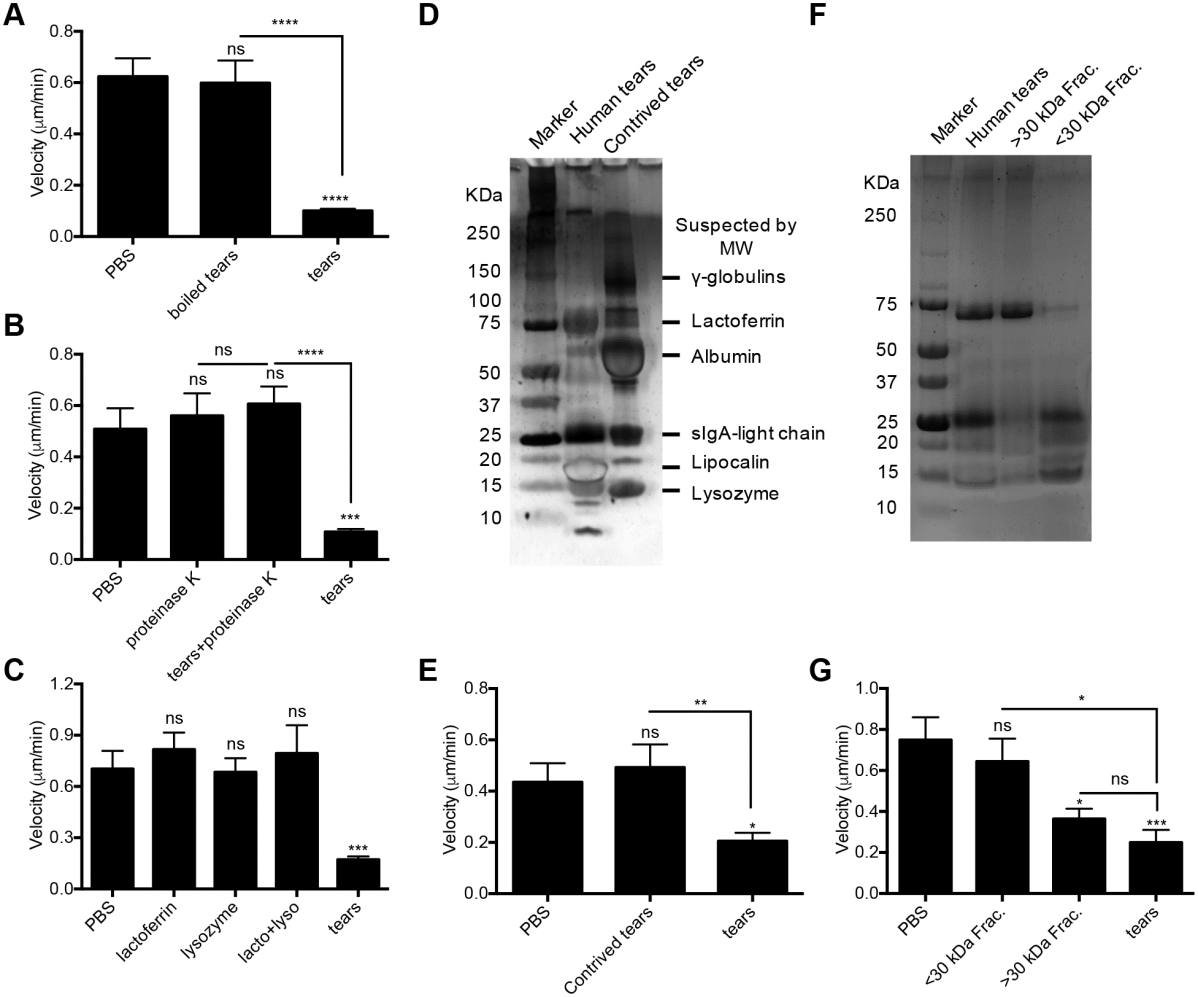
Heat-sensitive protein(s) in human tear fluid > 30 kDa inhibit *P*. *aeruginosa* twitching motility. *P*. *aeruginosa* PAO1 twitching velocity on twitching media was measured after 4 h. **(A)** Human tear fluid (25%) heated at 95°C for 10 min (boiled) then centrifuged to remove precipitated proteins lost inhibitory activity against twitching. **(B)** Human tear fluid (25%) treated by proteinase K (100 μg/mL) at 42°C for 2 h lost inhibitory activity. **(C)** Purified lysozyme (2 mg/mL), lactoferrin (2 mg/mL) or a combination cocktail do not inhibit *P*. *aeruginosa* PAO1 twitching motility. **(D)** Protein analysis of human tears and contrived tears by SDS-PAGE. **(E)** Contrived tears do not inhibit *P*. *aeruginosa* PAO1 twitching motility. **(F)** Protein analysis of human tear fluid fractions by SDS-PAGE after separation using a ~30 kDa cut-off column. **(G)** Tear fractions > 30 kDa inhibited PAO1 twitching motility. Data are shown as the mean ± SEM per sample from three independent experiments. Significance was determined using one-way ANOVA with Tukey's post-hoc analysis. ****, *P* < 0.0001; ***, *P* < 0.001; **, *P* < 0.01; *, *P* < 0.05; ns, not significant.

To begin to identify the tear factor(s) involved in twitching inhibition, human tear fluid was boiled or treated with proteinase K prior to bacterial exposure. In each instance, tears lost their inhibitory effects on twitching motility (Fig 5A and 5B) suggesting a heat-labile protein(s) was involved. Lysozyme and lactoferrin (alone or combined) at concentrations found in human tears [25] had no effect on twitching motility (Fig 5C). Commercially available contrived human tears containing lysozyme, lipocalin, albumin, lactoferrin, and gamma-globulins, and resembling tear fluid by SDS-PAGE (Fig 5D), also had no effect (Fig 5E). However, separation of human tear fluid into molecular weight fractions greater or less than ~30 kDa (Fig 5F), revealed that > ~30 kDa fractions inhibited twitching motility, while < ~30 kDa fractions did not (*P* < 0.05, ANOVA) (Fig 5G).

**Fig 6:**
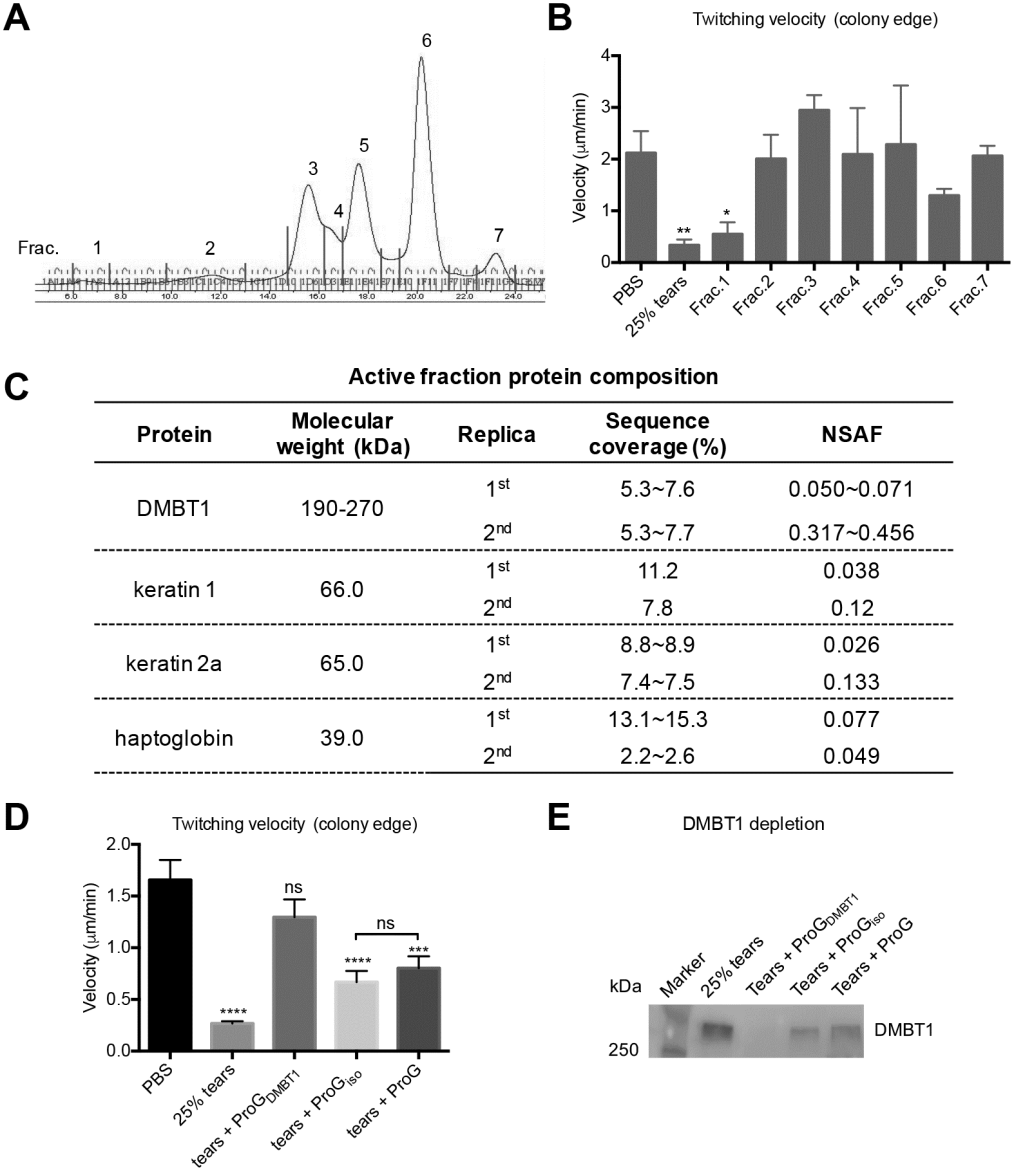
Identification of DMBT1 as the tear fluid inhibitor of *P*. *aeruginosa* twitching motility. **(A)** Human tear fluid was separated into 7 fractions using size exclusion chromatography. **(B)** Effect of tear fractions on twitching velocity of PAO1 reveals a high Mw fraction retains inhibitory activity. **(C)** Mass spectrometric analysis of high Mw tear fractions from two size-exclusion experiments reveal 4 proteins common to fractions inhibiting twitching motility. **(D)** DMBT1-depleted human tear fluid does not inhibit twitching motility of PAO1. **(E)** Western blot analysis of samples used in (D) shows depletion of DMBT-1 from tear fluid, and partial depletion by isotype control and protein G only beads control. Data shown in panels B and D as mean ± SEM from three independent experiments. Significance was determined using a one-way ANOVA with Tukey's post-hoc analysis. ****, *P* < 0.0001; ***, *P* < 0.001; **, *P* < 0.01; *, *P* < 0.05; ns, not significant.

Human tear fluid was separated into 7 fractions by size exclusion chromatography (Fig 6A & S2A Fig), and each fraction tested for inhibition of twitching motility in duplicate experiments. Consistent with our previous results (Fig 5F), only the high molecular weight fractions (Fraction 1 in Fig 6A, and Fraction 2 in S2A Fig) significantly inhibited *P*. *aeruginosa* PAO1 twitching motility (Fig 6B & S2B Fig). Since the active fraction in the first experiment (S2A & S2B Fig) was of high molecular weight, a different column material (Superose 6) was used for the second fractionation to obtain better protein separation. Proteins in the active fractions (Fraction 1 in Fig 6A, and Fraction 2 in S2A Fig) were analyzed by mass spectrometry. Results revealed the presence of only 4 proteins; DMBT1 (Deleted in Malignant Brain Tumors 1), keratin 1, keratin 2a and haptoglobin, were present in active fractions of both experiments (Fig 6C & S1 Table).

It has been shown that spectrum counting and mass spectrometry chromatograms correlate with quantitative changes in protein amount [26]. Considering that large proteins tend to contribute more peptide/spectra than small ones, the NSAF (normalized spectral abundance factor) was used to account for the effect of protein length on spectral counts, which allowed a comparison of individual protein abundance in multi-protein complexes [27]. Based on NSAF values, DMBT1 showed the highest relative abundance in active fractions for both experiments, and was therefore considered the most likely candidate for tear inhibition of *P*. *aeruginosa* twitching motility.

To directly evaluate DMBT1 involvement in tear inhibition of twitching motility, DMBT1 was immunoprecipitated from human tear fluid. Human tear fluid-depleted of DMBT1 lost inhibition of *P*. *aeruginosa* twitching motility compared to 25% tears, with no significant difference in twitching velocity found between PBS and tear-fluid depleted of DMBT1 (Fig 6D). It was noted that tear fluid treated with isotype control antibody and protein G only beads partially inhibited twitching motility (Fig 6D). Western immunoblot (Fig 6E) confirmed that DMBT1 was efficiently removed from tear fluid. Fig 6E also showed that the isotype control and protein G only beads partially depleted DMBT1 suggesting a degree of non-specific binding, but consistent with observed partial effects on twitching velocity (Fig 6D). Together, these data suggested that DMBT1 was required for human tear fluid inhibition of *P*. *aeruginosa* twitching motility.

### DMBT1 purified from saliva also inhibits *P*. *aeruginosa* twitching motility

DMBT1 is expressed in multiple tissues and body fluids and can undergo modifications that could affect its function at specific sites [28, 29]. Since DMBT1 is abundant in saliva, we tested if human saliva could inhibit *P*. *aeruginosa* twitching motility. Results confirmed a significant reduction in *P*. *aeruginosa* twitching velocity by human saliva treatment from a mean (± SEM) of 1.16 (± 0.14) μm/min in PBS controls to 0.27 (± 0.02) μm/min with human saliva treatment (*P* < 0.0001, one-way ANOVA, Tukey's post-hoc analysis). The latter velocity was not significantly different from the reduction achieved by 25% tears 0.48 (± 0.06) μm/min (*P* = 0.2316). Thus, DMBT1 was purified from human saliva, and tested for inhibition of twitching motility. DMBT1 purification was achieved by exploiting DMBT1 binding to/aggregation of *Streptococcus pyogenes*, and the bound DMBT1 then released from the aggregated *S*. *pyogenes* with EDTA treatment [30, 31]. Results (S3 Fig & S2 Table) showed that after purification using *S*. *pyogenes*, DMBT1 was the only protein common in two independent fractions (two experimental replicates), and the major protein in the purified fractions based on NSAF. This fraction was referred to as "purified DMBT1" in subsequent experiments.

**Fig 7:**
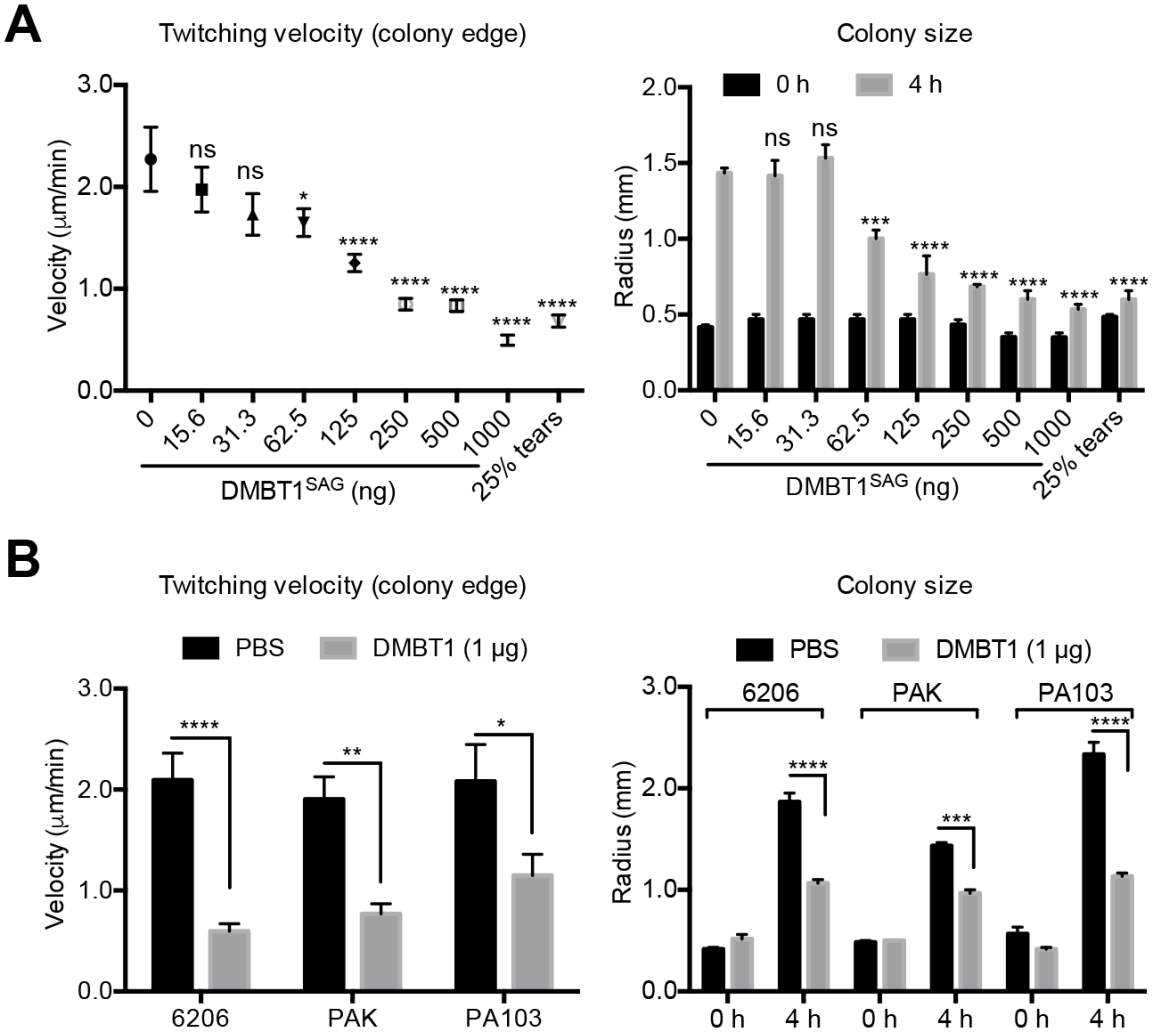
Saliva-purified DMBT1 inhibits twitching of multiple *P*. *aeruginosa* strains. DMBT1 solutions (5 μl) at different concentrations up to 200 ng/μl DMBT1 in PBS were dropped onto twitching media then inoculated with bacteria and incubated for 4 h at 37°C. Twitching velocity and colony size were quantified. **(A)** Purified DMBT1 from saliva inhibited *P*. *aeruginosa* PAO1 twitching velocity and colony size expansion in a dose-dependent manner. **(B)** Purified DMBT1 from saliva (1 μg) inhibited twitching velocity and colony size expansion in three other *P*. *aeruginosa* strains. In each panel, data are expressed as mean ± SEM per sample from three independent experiments. Significance was determined by one-way ANOVA with Tukey's post-hoc analysis for twitching velocity, and two-way ANOVA with Tukey's post-hoc analysis for colony size. ****, *P* < 0.0001; ***, *P* < 0.001; **, *P* < 0.01; *, *P* < 0.05; ns, not significant.

The purified DMBT1 fraction from saliva caused a dose-dependent inhibition of PAO1 twitching velocity and colony size expansion (Fig 7A). In each instance, a significant inhibition was achieved with concentrations of DMBT1 equal to or greater than 12.5 ng/μl (5 μl drop placed onto twitching media) (Fig 7A). DMBT1 at 1 μg in PBS (placed on twitching media) also inhibited twitching motility of all of three other *P*. *aeruginosa* strains (Fig 7B).

Mass spectrometry analysis of high Mw fractions that inhibit *P*. *aeruginosa* twitching motility, from human tear fluid (S1 Table) or saliva (S2 Table) showed that only DMBT1 was present in all samples, and that it was the most abundant protein further supporting the hypothesis that DMBT1 is responsible for inhibition of twitching motility in tear fluid and saliva.

### Saliva-purified DMBT1 inhibits *P*. *aeruginosa* traversal of multilayered human corneal epithelial cells *in vitro*

Our previous studies have shown that twitching motility contributes to *P*. *aeruginosa* traversal of corneal epithelial cells [19], and that tear fluid protects against *P*. *aeruginosa* traversal [8]. Thus, we hypothesized that purified DMBT1 would also inhibit *P*. *aeruginosa* traversal of multilayered human corneal epithelial cells.

**Fig 8:**
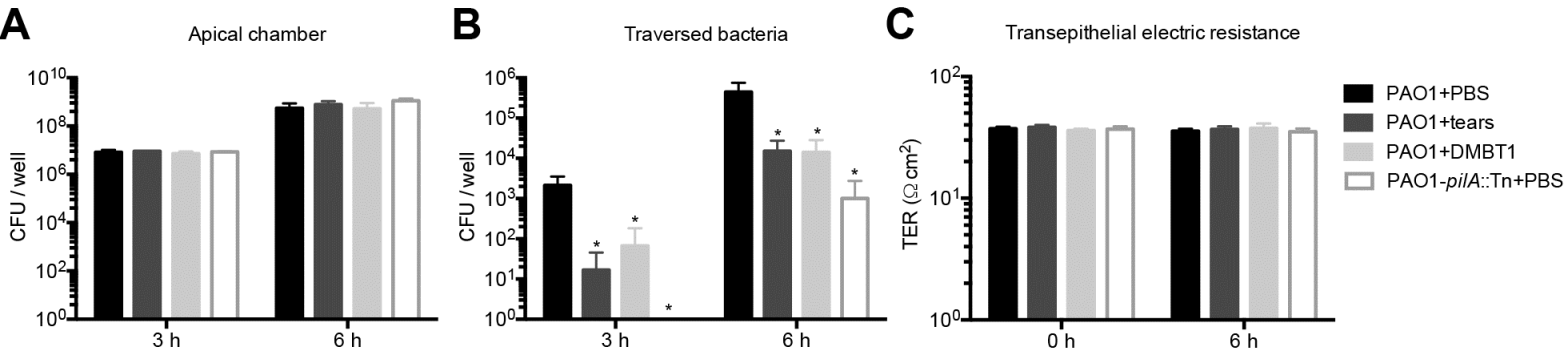
DMBT1 purified from saliva inhibits *P*. *aeruginosa* traversal of multilayered human corneal epithelial (hTCEpi) cells *in vitro*. Traversal of *P*. *aeruginosa* PAO1 or its *pilA* mutant across cultured airlifted human corneal epithelial cells *in vitro*. PAO1 was incubated in PBS, treated with 50% human tear fluid, or with 100 ng/μl of DMBT1. The *pilA* mutant was added in PBS. **(A)** Viable bacterial counts (means ± SD) in the apical chamber were determined at 3 and 6 h to evaluate bacterial growth. **(B)** Viable bacterial counts (means ± SD) in the basal chamber were determined at 3 and 6 h to estimate traversed bacteria. In each instance significance was determined using one-way ANOVA with Tukey’s post-hoc analysis. *, *P* < 0.05. **(C)** TER (Ω·cm^2^) across the human corneal epithelial cells over 6 h. A Transwell filter without cells was used as a control. TER values shown represent TER_(sample)_—TER_(bank)_.

Human corneal epithelial cells (hTCEpi) were grown on Transwell filters (3 μm pore-size) and airlifted for 7 days to form multilayers. *P*. *aeruginosa* PAO1 was added to the apical surface with DMBT1 solution, PBS or human tear fluid. After 3 and 6 h, viable bacteria from the apical and basal chambers were counted. Human tear fluid (50%) or DMBT1 (100 ng/μl) had no effect on bacterial growth in the apical chamber (Fig 8A), but significantly inhibited *P*. *aeruginosa* traversal at 3 h and 6 h (Fig 8B). As expected, the *pilA* mutant showed significantly reduced traversal compared to wild-type PAO1. Transepithelial resistance (TER) was unaffected in each sample over the 6 h incubation (Fig 8C) consistent with our published data for wild-type *P*. *aeruginosa* [19].

### DMBT1 purified from saliva protects against *P*. *aeruginosa* corneal infection *in vivo*

Our previous studies showed that twitching motility was important for *P*. *aeruginosa* virulence in a murine scarification model of corneal infection [18], and that human tear fluid can protect against *P*. *aeruginosa* corneal infection in both scarification and healing injury models [8]. Thus, we explored if DMBT1 could protect against *P aeruginosa* infection *in vivo* using a mouse model.

**Fig 9:**
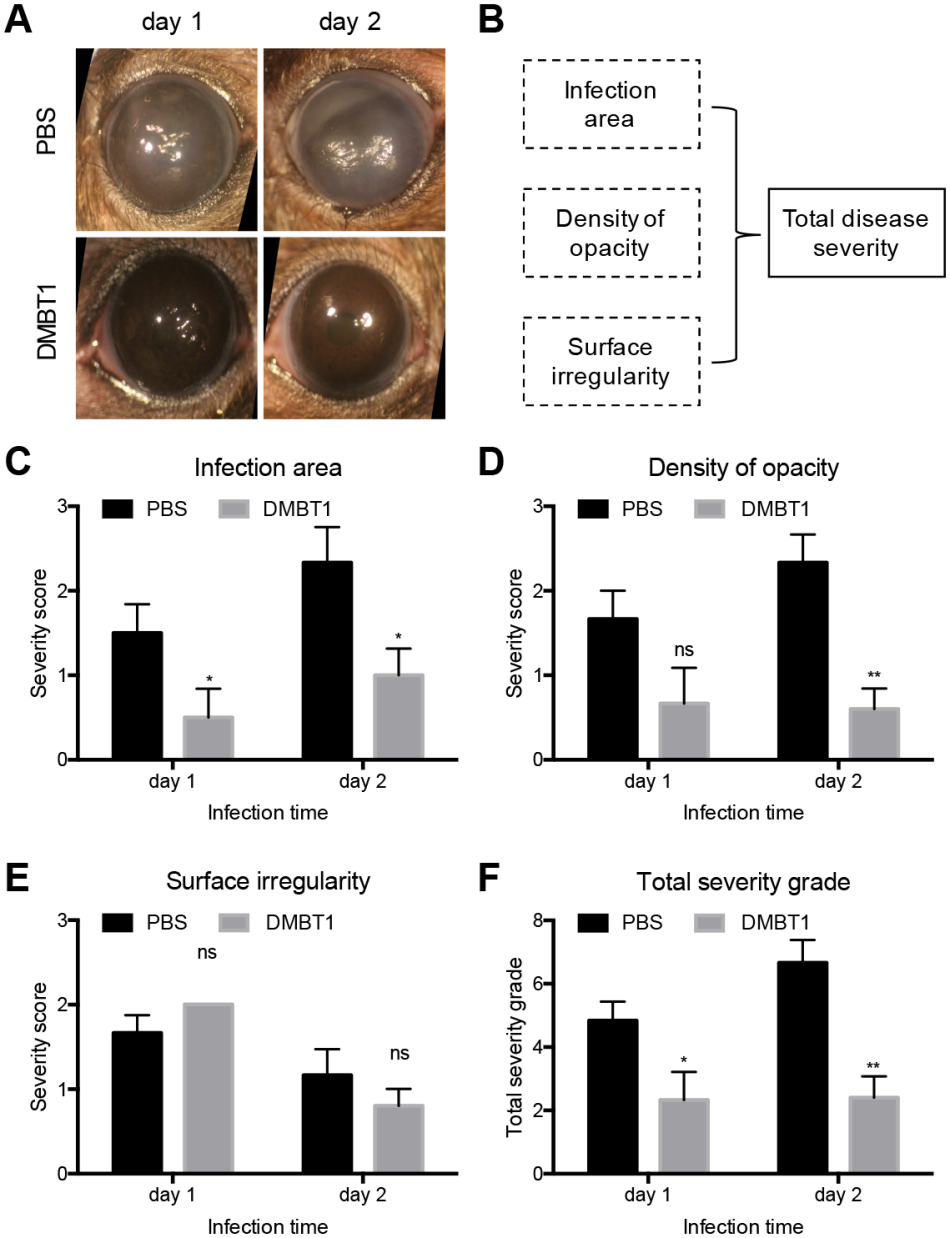
DMBT1 purified from saliva protects against *P*. *aeruginosa* corneal infection. **(A)** Representative images of C57BL/6 murine corneas at 24 and 48 h and post-infection with *P*. *aeruginosa* PAO1 in PBS or DMBT1 (150 ng/μL). **(B)** Schematic for grading disease severity of infected murine corneas. Effect of DMBT1 on corneal infection disease severity scores at 24 and 48 h comparing **(C)** area of infection, **(D)** density of opacity, **(E)** corneal surface irregularity, and (F) total disease severity, the sum of values shown in (C), (D), and (E). Data are reported as the mean ± SEM per group over three independent experiments (6 mice per group in total). Significance of differences between groups was determined using the Mann-Whitney U test. **, *P* < 0.01; *, *P* < 0.05; ns, not significant.

After scarification injury and 6 h healing, mouse corneas were inoculated with *P*. *aeruginosa* PAO1 in PBS or DMBT1 (see Methods). Representative images (Fig 9A) show that corneas inoculated with PAO1 in PBS presented with clear signs of infection after day 1 that progressed further by day 2. DMBT1 treated corneas showed reduced disease pathology at day 1, and greatly reduced pathology at day 2. Quantification of disease severity using a grading system that accounted for area of infection, density of opacity, and surface irregularity (Fig 9B) [32] showed DMBT1 treated corneas had a significant reduction in area of infection at day 1 and day 2 (Fig 9C), and in corneal opacity at day 2 (Fig 9D). Surface irregularity was relatively unaffected (Fig 9E). Overall disease severity was significantly reduced in DMBT1 treated eye at both time points (Fig 9F) showing that DMBT1 protected corneas from *P*. *aeruginosa* keratitis.

### DMBT1 does not affect *P*. *aeruginosa* PilA expression or cyclicAMP but does bind pili

Salivary DMBT1 is a well recognized agglutinin for Gram-positive and Gram-negative bacteria except *P*. *aeruginosa* [31, 33–36]. Tear fluid DMBT1 was also shown to bind *Staphylococcus aureus*, but not *P*. *aeruginosa*, using anti-DMBT1 antibody in a dot-immunoblot assay [29]. We confirmed that DMBT1 purified from saliva aggregates *S*. *pyogenes* [30], but does not aggregate *P*. *aeruginosa* (S4 and S5 Videos), suggesting a novel mechanism for inhibiting *P*. *aeruginosa* twitching motility.

**Fig 10:**
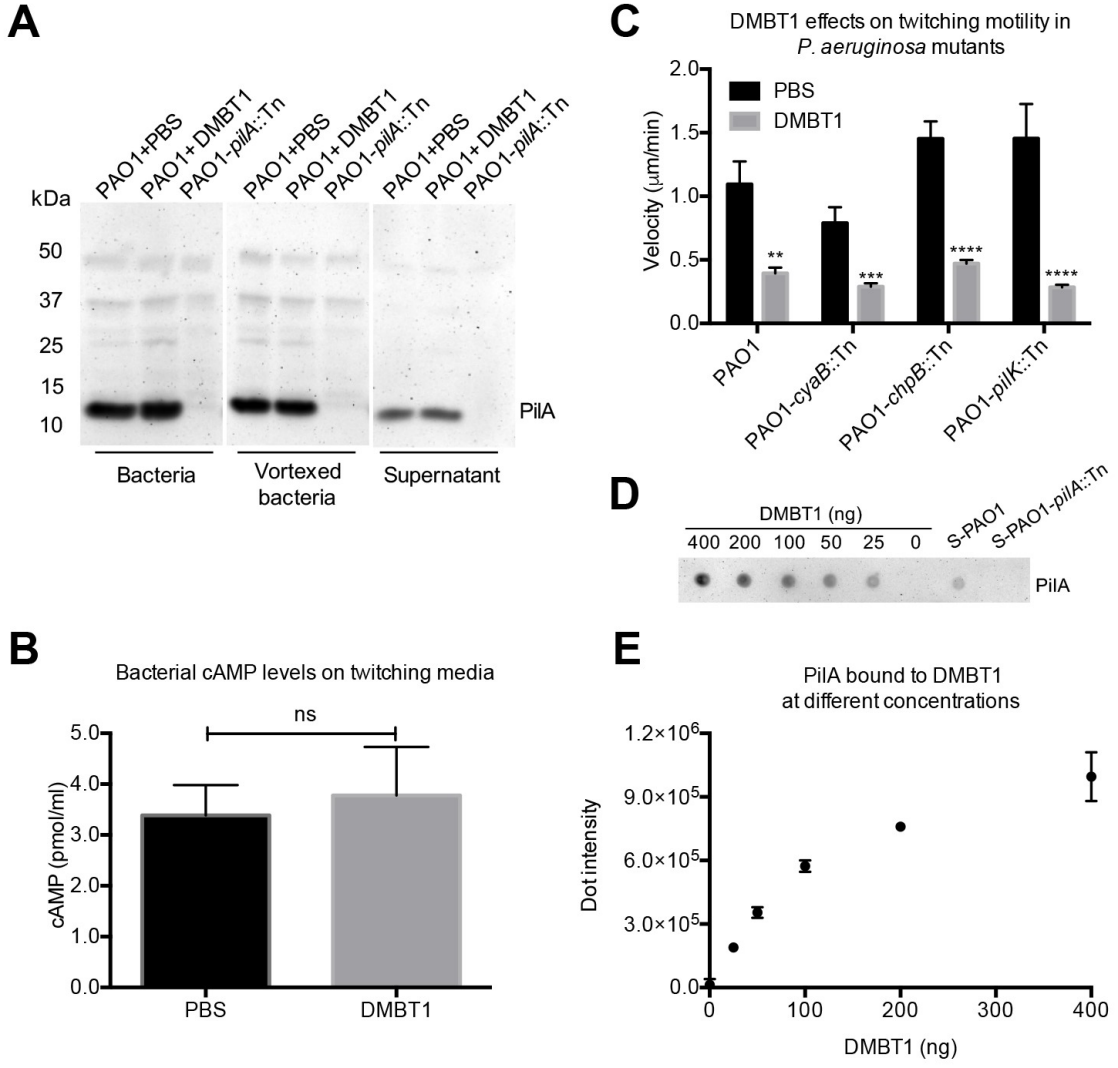
DMBT1 does not affect *P*. *aeruginosa* PilA expression or cyclicAMP levels, but inhibits twitching of PAO1 mutants in *cyaB*, *chpB*, and *pilK*. **(A)** Western immunoblot of PilA expression in *P*. *aeruginosa* PAO1 after DMBT1 (100 ng/μl) treatment on twitching media for 4 h. PilA protein levels were unaffected by DMBT1 exposure in all samples (bacteria, vortexed bacteria, and supernatant, see Methods). **(B)** CyclicAMP levels of *P*. *aeruginosa* PAO1 treated with DMBT1 (100 ng/μl) on twitching media for 4 h. **(C)** Effects of DMBT1 (100 ng/μl) on twitching velocity of PAO1 mutants in *cyaB*, *chpB* and *pilK* after 4 h. Data are shown as the mean ± SEM per sample from three independent experiments. Significance was determined using one-way ANOVA with Tukey's post-hoc analysis. ****, *P* < 0.0001; ***, *P* < 0.001; **, *P* < 0.01; *, *P* < 0.05; ns, not significant. **(D)** Dot-immunoblot assay using anti-PilA antibody to show the binding of PAO1 pili to DMBT1 after 40 min incubation with a pili-containing extract from PAO1. Diluted extracts from PAO1 (S-PAO1) or its *pilA* mutant (S-PAO1-*pilA*::Tn) (see Methods) were used as controls. A representative experiment of two independent experiments is shown. **(E)** Quantification of dot-intensity from the dot-immunoblot assay shown in D. Data are shown as the mean ± SEM of triplicate measurements from each sample.

Type IV pilus (T4P) production and twitching motility in *P*. *aeruginosa* is controlled by the Pil-Chp pathway (encoded by gene cluster IV) containing *pilG/H/I/J/K* and *chpA/B/C/D/E* genes [37]. The Chp system controls T4P production by modulation of cyclicAMP; twitching motility is cyclicAMP-independent [38]. To begin to explore the mechanism for DMBT1 inhibition of twitching motility, we examined some key elements of the pathways involved. Western immunoblots showed no difference in PilA expression by *P*. *aeruginosa* PAO1 after DMBT1 exposure for 4 h (Fig 10A) suggesting that pilin production was unaffected. Purified DMBT1 also had no effect on cyclicAMP levels in *P*. *aeruginosa* PAO1 collected from twitching media after 4 h (Fig 10B). Since many chemotaxis mutants of *P*. *aeruginosa* lose twitching motility, it is difficult to determine if DMBT1 inhibition of twitching motility involves chemotaxis genes [38]. However, the latter study identified three mutants of *P*. *aeruginosa* strain PAK that retained twitching motility; mutants in *cyaB* (encoding an adenylate cyclase to control cAMP synthesis), *chpB* (encoding a methylesterase) which can adjust the methylation status of the sensor module in the pili-mediated chemotaxis system [37], and *pilK* (encoding a methyltransferase) [38]. In the present study, *cyaB*, *chpB*, and *pilK* mutants of strain PAO1 also retained twitching motility under control conditions (Fig 10C). DMBT1 inhibited twitching of all three mutants (Fig 10C), suggesting that those genes are not needed for DMBT1-mediated twitching inhibition. Dot-immunoblot assays, however, showed that purified DMBT1 could bind pili extracted from *P*. *aeruginosa* strain PAO1 (Fig 10D and 10E), suggesting that the mechanism for inhibition of twitching motility involves a direct interaction with *P*. *aeruginosa* pili.

## Discussion

Previously, we showed that tear fluid could protect human corneal epithelial cells and mouse corneas against *P*. *aerugino*sa infection [8, 19], a protective activity mechanistically separable from bacteriostatic activity. Here, we explored the mechanisms involved and found that human tear fluid can inhibit surface-associated twitching motility by *P*. *aeruginosa*, dependent on the glycoprotein DMBT1. DMBT1, also known as GP340, is abundant in various mucosal fluids. Used alone, DMBT1 purified from saliva inhibited twitching motility, suggesting it is both necessary and sufficient for the protective activity of tear fluid. DMBT1 did not suppress *P*. *aeruginosa* viability, or aggregate bacteria, and its inhibition of twitching was not associated with changes in pilin expression or bacterial cyclicAMP. However, DMBT1 bound pili extracted from *P*. *aeruginosa* suggesting a direct interaction with pili is involved in twitching inhibition. Reflecting the known importance of twitching in virulence, DMBT1 protected multilayers of human corneal epithelial cells against *P*. *aeruginosa* traversal, and reduced disease severity in an *in vivo* animal model. Thus, this glycoprotein, or an active derivative, may have potential as a biocompatible therapeutic intervention against *P*. *aeruginosa* that acts without suppressing bacterial viability or bacterial aggregation. Although this study was focused on tear fluid and the cornea, the findings might be broadly applicable to other mucosal surfaces that *P*. *aeruginosa* infects given that DMBT1 is present in human saliva, the small intestine, and airways [28, 29].

DMBT1 was first isolated from saliva using affinity adsorption to *Streptococcus mutans*, and identified as a ~300–400 kDa glycoprotein [28]. It belongs to the scavenger receptor cysteine-rich (SRCR) protein superfamily, which is highly conserved down to sponges as secreted or membrane-bound proteins [39]. The human chromosome contains one copy of the gene encoding DMBT1 located on chromosome 10q26.13. However, there are different human DMBT1 alleles within the population and different isoforms in different tissues governed by alternative splicing and post-translation modification, e.g. DMBT1 variations include the number of SRCR domains or patterns of glycosylation [28, 39, 40]. Approximately 25% of the molecular mass of salivary-derived DMBT1^SAG^ (Salivary Agglutinin) is due to glycosylation (~10% for N-glycosylation, and ~15% for O-glycosylation) [41, 42]. Differences in DMBT1 glycosylation were reported between saliva-derived DMBT1^SAG^ and lung-derived DMBT1^GP340^, the latter lacking Lewis (Le) antigens [43]. Moreover, two isoforms of DMBT1 derived from human tear fluid expresses sialyl-Le^a^ antigens [44], not sialyl-Le^x^ antigens expressed by DMBT1^SAG^. In our studies, DMBT1 depletion from human tear fluid removed inhibition of twitching motility, while DMBT1 purified from saliva inhibited twitching motility. Thus, reported differences in sialyl-Le antigens do not affect this function suggesting that tear fluid and salivary DMBT1 isoforms share a common domain(s) to fulfill twitching inhibition. Detailed structure function studies to identify domains inhibiting twitching will require further investigation.

The SRCR domains play a key role in the function of DMBT1 in mucosal immunity as a bacterial agglutinin that binds many pathogens including Gram-positive and Gram-negative bacteria, and viruses [31, 33–36]. However, DMBT1 does not aggregate *P*. *aeruginosa* strain PAO1 [29], a finding confirmed in the present study (S4 and S5 Videos).

DMBT1 is known to bind other mucosal fluid antimicrobial and immune defense proteins including; SP-D [45], SP-A [46], lactoferrin [47] and secretory IgA [48]. Thus, some of its apparent activities can depend on its binding partners. However, no other known defense factors were found in the mass spectrometry analysis of the high Mw fractions of tear fluid or DMBT1 purified from saliva, which both inhibited twitching motility. While the active tear fraction containing DMBT1 did contain three other proteins in replicate experiments; keratin 1, keratin 2a, and haptoglobin (Fig 4C), none of them were present in the saliva-purified fractions containing DMBT1 that inhibited twitching.

That neither tear fluid nor purified DMBT1 inhibited the growth of *P*. *aeruginosa* strain PAO1 (Figs 2E and 8A) is consistent with our previously published data showing a lack of tear fluid bacteriostatic activity against many *P*. *aeruginosa* isolates [13].

Thus, the mechanism by which DMBT1 inhibits *P*. *aeruginosa* twitching motility appears to be independent of bacterial aggregation, known DMBT1 binding partners, other proteins present in the active fractions with DMBT1, and bacteriostatic activity. Our data also indicated that pilin expression, and bacterial cyclicAMP levels were unaffected by DMBT1 exposure, and DMBT1 could inhibit twitching motility of *P*. *aeruginosa* mutants in *pilK* (encoding a methyltransferase), *chpB* (encoding a methylesterase), or *cyaB* (encoding an adenylate cyclase), suggesting that none of these factors were involved.

Purified DMBT1 did, however, bind pili extracted from *P*. *aeruginosa* PAO1 suggesting that twitching motility inhibition involves direct interaction with pili. Such interactions could affect numerous aspects of pilus function including; their extension or retraction, their interactions with surfaces (biotic and abiotic), or cause their aggregation. However, twitching motility could also be compromised by DMBT1 at other levels including an alteration of gene expression in the Pil-Chp pathway (or its regulation), or interfering with small molecule regulation, e.g. cyclic-di-GMP [21, 22]. Targets for future study could include pilus extension or retraction motor proteins, e.g. ATPases PilB or PilT respectively [49, 50], or the chemosensory protein PilJ which directly interacts with PilA [23], and also controls pilus extension [51].

It is possible that DMBT1 interacts with other *P*. *aeruginosa* surface antigens or structures, in addition to pili, that affect twitching motility and/or other bacterial functions. While DMBT1 did not affect bacterial swimming motility (S5 Video), the full spectrum of DMBT1-*P*. *aeruginosa* interactions, and their consequences, will require further investigation.

The protective mechanism of DMBT1 in our *in vitro* and *in vivo* infection models is likely to involve suppression of twitching motility, given that twitching is critical to pathogenesis in the cornea [18]. While enabling bacteria to traverse surface epithelial cells, twitching may be used for trafficking along/through the basal lamina [52], and/or for disseminating within the underlying corneal stroma [53]. Twitching is also important for biofilm formation [54], a key virulence determinant when infection is device-related [55, 56].

However, activities of DMBT1 other than inhibition of twitching might have contributed to its protective activities in our *in vivo* experiments, for example through binding and association with other tear defense proteins. Surfactant protein D (SP-D) which readily binds DMBT1 can protect corneal epithelial cells against *P*. *aeruginosa* invasion [57], and it contributes to clearing *P*. *aeruginosa* from the ocular surface [58]. IgA, another binding partner, can prevent *P*. *aeruginosa* binding to mouse corneas and reduces severity of *P*. *aeruginosa* keratitis [59]. Both factors can also function as opsonins facilitating phagocytosis and clearance of *P*. *aeruginosa* [60, 61]. Also possible, is that DMBT1 influences pathogenesis via direct effects on resident or infiltrating host cells. Indeed, it can stimulate a dose-dependent chemokinesis (random migration) of alveolar macrophages, suggesting role(s) in respiratory inflammatory and immune responses [46]. It is also able to activate classical and lectin pathways of the complement system [62, 63]. Teasing apart the relative contributions of different DMBT1 activities in protecting the cornea against *P*. *aeruginosa* infection *in vivo* will not be straightforward.

Mice also express a homolog of DMBT1 [64]. The ability of *P*. *aeruginosa* to infect control eyes in our study likely reflects characteristics of the infection model, in which murine corneas were washed with PBS prior to bacterial inoculation, and mice sustained under anesthesia for 4 h after inoculation. This methodology would remove murine tear fluid, and reduce tear flow, likely compromising the ability of murine DMBT1 to exert protective effects.

In sum, the results of this study suggest that DMBT1 inhibition of twitching motility contributes to mechanisms by which mucosal fluids protect against *P*. *aeruginosa* infection, and is likely accomplished by direct binding to pili. Twitching motility, important to *P*. *aeruginosa* virulence both *in vitro* and *in vivo*, is also key to biofilm formation. Thus, discovery that DMBT1 modulates bacterial virulence factor expression adds to our understanding of how mucosal fluids defend tissue surfaces against infection. Further, DMBT1 or its derivatives may hold promise for development of biocompatible strategies for preventing *P*. *aeruginosa* infection that act by altering expression of virulence genes rather than agglutinating bacteria, or suppressing their viability. Whether wearing a contact lens or other device at mucosal surfaces impacts the quantity, location, or integrity of DMBT1, and if any changes relate to pathogenesis of infection, remains to be determined.

## Materials and methods

### Ethics statement

Human tear fluid and saliva were collected from healthy volunteers under a protocol approved by the Committee for the Protection of Human Subjects, University of California Berkeley. Informed, written consent was obtained from all participants. All procedures involving mice were carried out in accordance with standards established by the Association for the Research in Vision and Ophthalmology, under the protocol AUP-2016-08-9021 approved by the Animal Care and Use Committee, University of California Berkeley, an AAALAC accredited institution. The protocol adheres to PHS policy on the humane care and use of laboratory animals, and the guide for the care and use of laboratory animals. Anesthesia was achieved by intraperitoneal injection of an anesthetic cocktail containing ketamine (80 mg/Kg) and xylazine (10 mg/Kg), or ketamine (50 mg/Kg) and medetomidine (0.75 mg/Kg) for sustained anesthesia. Euthanasia was performed using carbon dioxide inhalation.

### Bacterial strains and culture conditions

*P*. *aeruginosa* strains PAO1, PAK, PA103 and 6206 were used. Bacteria were grown on tryptic soy agar (TSA) plates at 37°C for 16 h to obtain lawn cultures. *P*. *aeruginosa* PAO1 transposon insertion mutant *pilA*::Tn (PW8621) [65] lacking twitching motility, was grown on TSA with 60 μg/mL tetracycline, and used as a negative control. *P*. *aeruginosa* PAO1 transposon insertion mutants *cyaB*::Tn (PW6387), *chpB*::Tn (PW1760) and *pilK*::Tn (PW1757) [65] were also grown on TSA with 60 μg/mL tetracycline. Each of the transposon mutants was verified by PCR (S1 Fig) using previously reported primers [65] or primers designed for *pilK*. For twitching motility assays, bacteria were grown on twitching motility Gellan Gum media (TMGG, 0.8 g Gellan gum, 0.4 g tryptone, 0.2 g yeast extract, 0.2 g NaCl, 0.1 g MgSO_4_·7H_2_O, in 100 mL H_2_O) at 37°C in a humidified chamber for different times. *Streptococcus pyogenes* (ATCC19615) was grown in Brain and Heart Infusion (BHI) broth at 37°C overnight and used for purification of DMBT1.

### Reagents

Tear fluid was collected using a 30 μl volume capillary tube. Subjects were non-contact lens wearers, males and females, between 18 and 45 years of age, and with no ocular infection or inflammation at the time of collection. Approximately 5% of the tear fluid from each subject was plated on TSA to control for bacterial contamination; sterile tear fluid was pooled (from 6 to 8 subjects) and stored at -80°C until used. In different experiments, human tear fluid (25%) was boiled at 95°C for 10 min to denature heat-liable components, treated with proteinase k (Sigma-Aldrich, 100 μg/mL) at 42°C for 2 h, or fractionated using sterile water pre-rinsed Microcon centrifugal filter devices with membrane cutoffs of ~30 kDa (Millipore). Saliva was obtained from healthy volunteers, clarified by centrifugation at 3,800 x g for 10 min then used for testing its effect on twitching motility, and for DMBT1 purification as described below. Purified lactoferrin and lysozyme from human milk (2 mg/mL in PBS) were purchased from Sigma-Aldrich. Contrived tear fluid containing lysozyme, albumin, and *γ*-globulins was purchased from Ursa BioScience (MD, USA).

### Twitching motility assays

Twitching motility was measured using a method modified from the microscope slide assay described previously [66]. Bacteria were grown on TSA plates (supplemented with tetracycline if needed) at 37°C for 16 h. Twitching motility Gellan Gum media was dried for 20 min in a sterile airflow (BSL2 Biosafety Cabinet) before use and then 5 μL of tear fluid, PBS or other solution was dropped onto the twitching media until completely absorbed. Bacteria grown on TSA were collected, and mixed using a plastic inoculation loop and subsequently inoculated onto the twitching media using a sterile toothpick to achieve a tip-sized inoculum. A glass coverslip was gently placed onto the twitching media to create an interstitial space. The slides were then incubated at 37°C for 4 h unless otherwise stated. After indicated incubation times, 5 min time-lapse videos were captured at 10 s intervals via differential interference contrast (DIC) microscopy using a Nikon ECLIPS Ti microscope with a 60× oil-immersion objective at 37°C.

### Quantification of twitching motility

Twitching motility was quantified with three different methods. Firstly, individual bacterial twitching motility was quantified as the degree to which they modulated light in a DIC image. To normalize the contrast of each image and eliminate contrast bias in areas of high focus/illumination, a band pass filter (2–40 pixels) was used in ImageJ. The degree of modulation was measured as the standard deviation of intensity of each pixel during the length of the movie. The standard deviation map was then thresholded so that only regions containing bacteria were analyzed. A histogram of the standard deviation map was then used to measure the distribution of bacterial motility in each sample. A notched boxplot was used to represent each result. In a notched box plot, if the notches do not overlap then the distributions are significantly different. Secondly, twitching velocity was measured as the twitching distance of the colony leading edge divided by time. The bacterial distance traveled in a 5 min video was measured from location in the first slide to location in the last slide using ImageJ. Different treatment groups for bacteria were done in triplicate and ten bacteria were tracked in each video. Thirdly, bacterial colony size was measured soon after inoculation onto twitching media (time zero) and after different incubation times.

### Bacterial growth in the presence of tear fluid

*P*. *aeruginosa* PAO1 bacteria were grown on TSA media overnight and then diluted to OD_600_ 0.03 by use of TSB (tryptic soy broth) media. One microliter of diluted bacteria was dropped onto twitching media absorbed with PBS or 25% human tear fluid and incubated at 37°C for up to 24 h. After collection and serial dilution in PBS, samples were plated onto TSA agar and incubated at 37°C overnight to determine numbers of viable bacteria expressed as Colony Forming Units (CFU). Experiments were repeated five times.

### Size-exclusion chromatography of human tear fluid

Size-exclusion chromatography was performed on an AKTAmicro system using a Superdex 200 10/300 GL column (GE Healthcare) in the first separation, or a Superose 6 10/300 GL column (GE Healthcare) in the second separation, equilibrated in PBS (pH 7.4). To minimize peak broadening, short lengths of 0.15 mm i. d. tubing were used between the injection valve and the fraction collector. Human tear fluid was injected onto the column, and fractions of 250 μl were collected. Protein was detected by UV absorbance at 280 nm. Eluted fractions were pooled according to protein peaks and concentrated using a ~3 kDa cut-off filter (Millipore). The activity of eluted fractions against *P*. *aeruginosa* twitching motility was then assessed as described above.

### Mass spectrometry

Mass spectrometry (MS) was performed at the Proteomics/Mass Spectrometry Laboratory, University of California, Berkeley. A nano LC column was packed in a 100 μm inner diameter glass capillary with an emitter tip. The column consisted of 10 cm of Polaris C18 5 μm packing material (Varian, Agilent, CA), followed by 4 cm of Partisphere 5 SCX (Whatman, Sigma-Aldrich, MO). The column was loaded by use of a pressure bomb and washed extensively with buffer A (see below). The column was then directly coupled to an electrospray ionization source mounted on a Thermo-Fisher LTQ XL linear ion trap mass spectrometer. An Agilent 1200 HPLC equipped with a split line to deliver a flow rate of 300 nl/min was used for chromatography. Peptides were eluted using a 4-step MudPIT procedure [67]. Buffer A was 5% acetonitrile/ 0.02% heptaflurobutyric acid (HFBA); buffer B was 80% acetonitrile/ 0.02% HFBA. Buffer C was 250 mM ammonium acetate/ 5% acetonitrile/ 0.02% HFBA; buffer D was same as buffer C, but with 500 mM ammonium acetate.

Protein identification and quantification were done with Integrated Proteomics Pipeline (IP2, Integrated Proteomics Applications, Inc. San Diego, CA) using ProLuCID/Sequest, DTASelect2 and Census [68–70]. Tandem mass spectra were extracted into ms1 and ms2 files from raw files using RawExtractor [71], and searched against the human protein database plus sequences of common contaminants, concatenated to a decoy database in which the sequence for each entry in the original database was reversed [72].

### Immunoprecipitation of DMBT1

Immunoprecipitation was performed using mouse monoclonal anti-DMBT1 antibody (ab17779, Abcam, MA), or a mouse IgG1 isotype control (Thermo Fisher, NY), and protein G-magnetic beads (New England BioLabs, MA) according to manufacturer’s protocols. After coating protein G magnetic beads with either DMBT1 antibody or isotype control in a 20 μl reaction, the complex was incubated with 25 μl of 25% tears for 60 min at 4°C. Non-coated protein G magnetic beads were also used as a negative control. The supernatant was then collected for assessing its activity on twitching motility, and its DMBT1 protein content by Western immunoblotting (anti-DMBT1 antibody was diluted 1:1000). The beads were washed 3 times with PBS and then eluted with 20 μl of 0.1 M glycine (pH 2.5) for 3 min at room temperature twice. The supernatant was collected and neutralized with 2 M tris (pH 9.0). The proteins bound to beads were analyzed by MS as described previously.

### Purification of DMBT1 from human saliva

DMBT1 was purified from human saliva rather than tear fluid because saliva is more abundant and easier to collect. Purification of DMBT1 was performed as described previously [31, 73]. Briefly, clarified saliva was diluted 50% with PBS. *Streptococcus pyogenes* was incubated in BHI broth overnight at 37°C, collected by centrifugation at 3,800 x g for 5 min, and washed three times with PBS. Bacterial concentration was adjusted to ~5 x10^9^ CFU/mL. Equal volumes of bacterial suspension and diluted saliva were then mixed and incubated at 37°C for 60 min. Bacterial cells were collected again by centrifugation at 3,800 x g for 5 min, and washed three times with PBS. PBS (1.5 mL) containing 5 mM EDTA was then used to release bound protein at room temperature for 5 min. The bacterial culture was centrifuged at 15,000 x g for 5 min, the supernatant filtered using a 0.22 μm filter, and then dialyzed (Slide-A-Lyzer dialysis cassettes, Thermo Fisher, NY) against PBS at 4°C overnight. Dialyzed eluate was subjected to gel filtration chromatography on a Superose 6 10/300 GL column (GE Healthcare, CA) equilibrated in PBS (pH 7.4). The eluate at void volume was collected and used as purified DMBT1 from saliva. The presence and purity of DMBT1 was verified by mass spectrometry as described above. DMBT1 concentration was measured using a micro BCA protein assay kit (Thermo Scientific, IL, USA).

### Bacterial traversal assay

Telomerase-immortalized human corneal epithelial cells (~ 6 × 10^4^ cells) were seeded onto 24-well polyester tissue culture treated Transwell^™^ inserts (3 μm pore size, Corning Costar, NY) in KGM-2 medium containing 1.15 mM CaCl_2_ for 7 days, then airlifted for 7 days as previously described [74]. *P*. *aeruginosa* strain PAO1, or its *pilA* mutant (~1.3 × 10^6^ CFU) was inoculated on the apical surface of the cells in PBS, human tear fluid (50% in PBS) or DMBT1 (100 ng/μl in PBS) for 6 h at 37°C (5% CO_2_). Transepithelial resistance (TER) (Ω·cm^2^) was measured using an Epithelial Voltohmeter (World Precision Instruments, Inc., FL) before inoculating the bacteria, and after 6 h incubation. Transwell^™^ inserts without corneal cells served as negative controls. After 3 and 6 h, bacterial viable counts in the apical and basal chambers were determined to measure bacterial growth and epithelial traversal.

### Murine corneal infection

All procedures were approved by the University of California, Berkeley Animal Care and Use Committee. The scarification with healing murine model of corneal infection was used as previously described [8] with minor modification. Briefly, C57BL/6 mice (6 to 12 weeks old) were anesthetized by intraperitoneal injection of an anesthetic cocktail containing ketamine (80 mg/Kg) and xylazine (10 mg/Kg). Eyes were checked for corneal clarity using a stereomicroscope prior to the initiation of experiments. Three parallel scratches were made on the right cornea of each anesthetized animal using a sterile 25 5/8-gauge needle. Mice were checked every 15 min until they woke up. After 6 h of epithelial healing, mice were anesthetized with a cocktail containing ketamine (50 mg/Kg) and medetomidine (0.75 mg/Kg), 70 μl per 25 g of body weight. Healing corneas were then washed with PBS (500 μl), then inoculated with 5 μl of a *P*. *aeruginosa* PAO1 suspension containing ~2 ×10^3^ CFU bacteria in DMEM mixed with either PBS or 200 ng/μl of DMBT1 at a ratio of 1:3. After 4 h infection under sustained anesthesia, an anesthesia reversal agent, atipamezole (3.75 mg/Kg), 50 μl per 25 g of body weight, was used to wake the mice. Mice were observed daily and ocular images were captured at 24 and 48 h post-inoculation using 2–3% isoflurane in oxygen inhalation for anesthesia. Corneal disease severity was graded by a masked observer using a previously described scoring system [32], which assesses area of infection, density of opacity, surface regularity and overall disease severity.

### Immunoblot assays

To study the effects of DMBT1 on pilin expression, PilA was measured by Western immunoblot using sample preparation methods based on previous studies [75]. PAO1 or its *pilA* mutant were treated with PBS or DMBT1 (100 ng/μl) as described above in the twitching motility assays section. After 4 h incubation, bacteria were washed from the twitching media and cover-slip with 50 mM Na_2_CO_3_ (pH 9.6). Bacterial OD_600_ was adjusted to 0.6, and 300 μl of bacterial culture centrifuged at 13,000 x g for 5 min, and re-suspended into SDS-PAGE sample buffer ("bacteria" sample). Another 300 μl of bacterial culture was extensively vortexed for 3 min to remove pili, centrifuged at 15,000 x g for 20 min, and the pellet dissolved in SDS-PAGE sample buffer ("vortexed bacteria" sample). The supernatant was placed at 4°C overnight, after adding MgCl_2_ to a concentration of 100 mM, and the next day centrifuged at 15,000 x g for 20 min. The pellet was dissolved in same volume of SDS-PAGE sample buffer ("supernatant" sample). All samples were heated at 95°C for 10 min, separated by SDS-PAGE (20% gel), and probed with antibody to PilA (1:5000) (a kind gift from Dr. Joanne Engel, University of California, San Francisco), then goat anti rabbit-HRP antibody (1:5000, Abcam, MA).

Dot-immunoblotting was used to test if DMBT1 could bind *P*. *aeruginosa* pili. Briefly, to prepare an extract of pili, a suspension of *P*. *aeruginosa* PAO1 in PBS was prepared to an OD_600_ of ~10. The suspension was vortexed for 3 min, centrifuged at 15,000 x g for 20 min, and the supernatant collected. MgCl_2_ solution (1 M) was added to the supernatant to a final concentration of 100 mM, and the supernatant placed at 4°C overnight. After centrifugation at 15,000 x g for 20 min, the pellet was resuspended in PBS (500 μl) to form a pili-containing extract. The same method was used to prepare a negative control extract of PAO1-*pilA*::Tn. For dot-immunoblot assays, 2 μl of DMBT1 in PBS (400 ng/μl and serial dilutions in PBS) were spotted onto a nitrocellulose membrane (0.2 μm pore-size, BioRad), along with a PBS control, the pili-containing extract from PAO1 (positive control), and the extract from the *pilA* mutant (negative control). The extracts were diluted 1 in 500 in PBS for use as controls. After the membrane was dry, it was blocked with 5% BSA for 1 h at room temperature, then washed with PBS for 5 min. The membrane was then incubated with the original (undiluted) pili-containing extract of PAO1 for 40 min at room temperature, then washed 5 times with PBS. Membranes were then probed with anti-PilA primary antibody (1:5000) and Goat anti-Rabbit HRP-conjugated secondary antibody (1:5000). Dot intensity was measured using AlphaView FluoChem HD2 software.

### CyclicAMP assay

Intracellular cyclicAMP of *P*. *aeruginosa* was measured as described previously [38] with minor modification. PAO1 was incubated on twitching media with DMBT1 (100 ng/μl) or PBS at 37°C for 4 h as described above in twitching motility assays. Bacteria were washed from twitching media with 0.9 M NaCl at 4°C, made with superpure water from Cayman Chemical (Ann Arbor, MI) and kept on ice. Bacterial suspensions were adjusted to the same OD_600_ value of ~1.0. Two equal volumes of each suspension were centrifuged at 13,000 x g for 2 min at 4°C, and the bacterial pellets washed twice with 1 mL of cold 0.9 M NaCl (final OD_600_ ~ 2.5). Bacterial pellets were suspended in 200 μl of 0.1 N HCl (made with superpure water) and incubated on ice for 10 min with occasional vortexing to lyse the bacteria. Lysates were centrifuged at 13,000 x g for 5 min at 4°C to remove cellular material, and the supernatant was assayed for cAMP concentration using a Cyclic AMP EIA Kit (Cayman Chemical) following the manufacturer’s protocol for sample acetylation.

### Statistical analysis

Data were expressed as a mean ± standard error of mean (SEM) unless otherwise stated. The significance of differences between groups was assessed by one or two-way ANOVA with Tukey's post-hoc analysis, or using the Mann-Whitney U test for *in vivo* experiments. P values of less than 0.05 were considered significant.

## Supporting information

S1 Video *P*. *aeruginosa* PAO1 twitching motility in PBS. Represents a 5 min time-lapse movie of *P*. *aeruginosa* twitching motility captured at an interval of 10 s with a 60 × oil-immersion lens. Scale bar = 20 μm. Frame rate = 10 fps.
(AVI)

S2 Video Human tear fluid inhibition of PAO1 twitching motility. Represents a 5 min time-lapse movie of *P*. *aeruginosa* twitching motility captured at an interval of 10 s with a 60 × oil-immersion lens. Scale bar = 20 μm. Frame rate = 10 fps.
(AVI)

S3 Video A twitching motility negative *pilA* mutant PAO1*-pilA*::Tn. Represents a 5 min time-lapse movie of *P*. *aeruginosa* twitching motility captured at an interval of 10 s with a 60 × oil-immersion lens. Scale bar = 20 μm. Frame rate = 10 fps.
(AVI)

S4 Video *P*. *aeruginosa* PAO1 movement in PBS at 37°C for 4 h. Represents a 10 s time-lapse movie of *P*. *aeruginosa* movement captured at no delay with a 40 × lens. Frame rate = 10 fps.
(AVI)

S5 Video PAO1 movement in purified DMBT1 (100 ng/μl) at 37°C for 4 h. Represents a 10 s time-lapse movie of *P*. *aeruginosa* movement captured at no delay with a 40 × lens. Frame rate = 10 fps.
(AVI)

S1 Fig PCR verification of *P*. *aeruginosa* PAO1 transposon insertion mutants. PAO1-*pilA*::Tn (PW8621), PAO1-*cyaB*::Tn (PW6387), and PAO1-*chpB*::Tn (PW1760) were verified by PCR with primers provided by the insertion mutant library database. PAO1-*pilK*::Tn (PW1757) was verified by PCR with the following primers; *pilK* flanking primers pilK-F (5'-AGATGCGCAACTCGGTATCC-3') and pilK-R (5'-TTCAGGGTTTCGGCGATCTC-3'). The red square was used to label target products.
(TIF)

S2 Fig **(A)** Fractions of human tear fluid separated by size exclusion chromatography (first experiment). **(B)** Effect of tear fractions on *P*. *aeruginosa* PAO1 twitching velocity measured in 5 min videos of each sample. Data are expressed as the mean ± SEM per sample from three independent experiments. Significance was determined using one-way ANOVA with Tukey's post-hoc analysis. **** P < 0.0001, ***P < 0.001.
(TIF)

S3 Fig DMBT1 purification from human saliva. **(A)** SDS-PAGE with silver stain (left panel) suggested DMBT1 was present after *S*. *pyogenes* treatment, and was confirmed by Western immunoblot (right panel) using anti-DMBT1 antibody. **(B)** and **(C)** Two independent experiments each showing that size-exclusion chromatography after DMBT1 purification from human saliva using *S*. *pyogenes* generated a high Mw fraction (fraction 1). Proteins were separated from aggregated *S*. *pyogenes* in human saliva using EDTA (5 mM). **(D)** Mass spectrometric analysis of fraction 1 after DMBT1 purification from saliva revealed the presence of DMBT1 in two independent experiments.
(TIF)

S1 Table Mass spectrometry results of human tear fluid fractions that inhibited twitching motility of *P*. *aeruginosa* PAO1. Results shown for two independent fractionations of human tear fluid using size-exclusion chromatography.
(TIF)

S2 Table Mass spectrometry results of two independent fractions obtained by size-exclusion chromatography after DMBT1 purification from saliva using *S*. *pyogenes*. Each fraction inhibited twitching motility of *P*. *aeruginosa* PAO1. Results represent two independent experiments.
(TIF)
