# Correction: Mucosal fluid glycoprotein DMBT1 suppresses twitching motility and virulence of the opportunistic pathogen *Pseudomonas aeruginosa*

**DOI:** 10.1371/journal.ppat.1006612

**Published:** 2017-09-12

**Authors:** Jianfang Li, Matteo M. E. Metruccio, Benjamin E. Smith, David J. Evans, Suzanne M. J. Fleiszig

Dr. Benjamin E. Smith should be included in the author byline instead of the Acknowledgments. He should be listed as the third author, and his affiliation is 4: the Graduate Group in Vision Science, University of California, Berkeley, California, United States of America. The contributions of this author are as follows: Data curation, Formal analysis, Methodology, Visualization, Writing—original draft, Writing—review & editing.

The correct citation is: Li J, Metruccio MME, Smith BE, Evans DJ, Fleiszig SMJ (2017) Mucosal fluid glycoprotein DMBT1 suppresses twitching motility and virulence of the opportunistic pathogen *Pseudomonas aeruginosa*. PLoS Pathog 13(5): e1006392. https://doi.org/10.1371/journal.ppat.1006392

The following information is missing from the Funding section: This study was supported by the National Institutes of Health (P30 EY003176, BES).
