# Supplementary figures and images for: Mucosal fluid glycoprotein DMBT1 suppresses twitching motility and virulence of the opportunistic pathogen Pseudomonas aeruginosa
Source: PLoS Pathog. 2017 May 10;13(5):e1006392. doi: 10.1371/journal.ppat.1006392 (PMC5440049; doi:10.1371/journal.ppat.1006392)

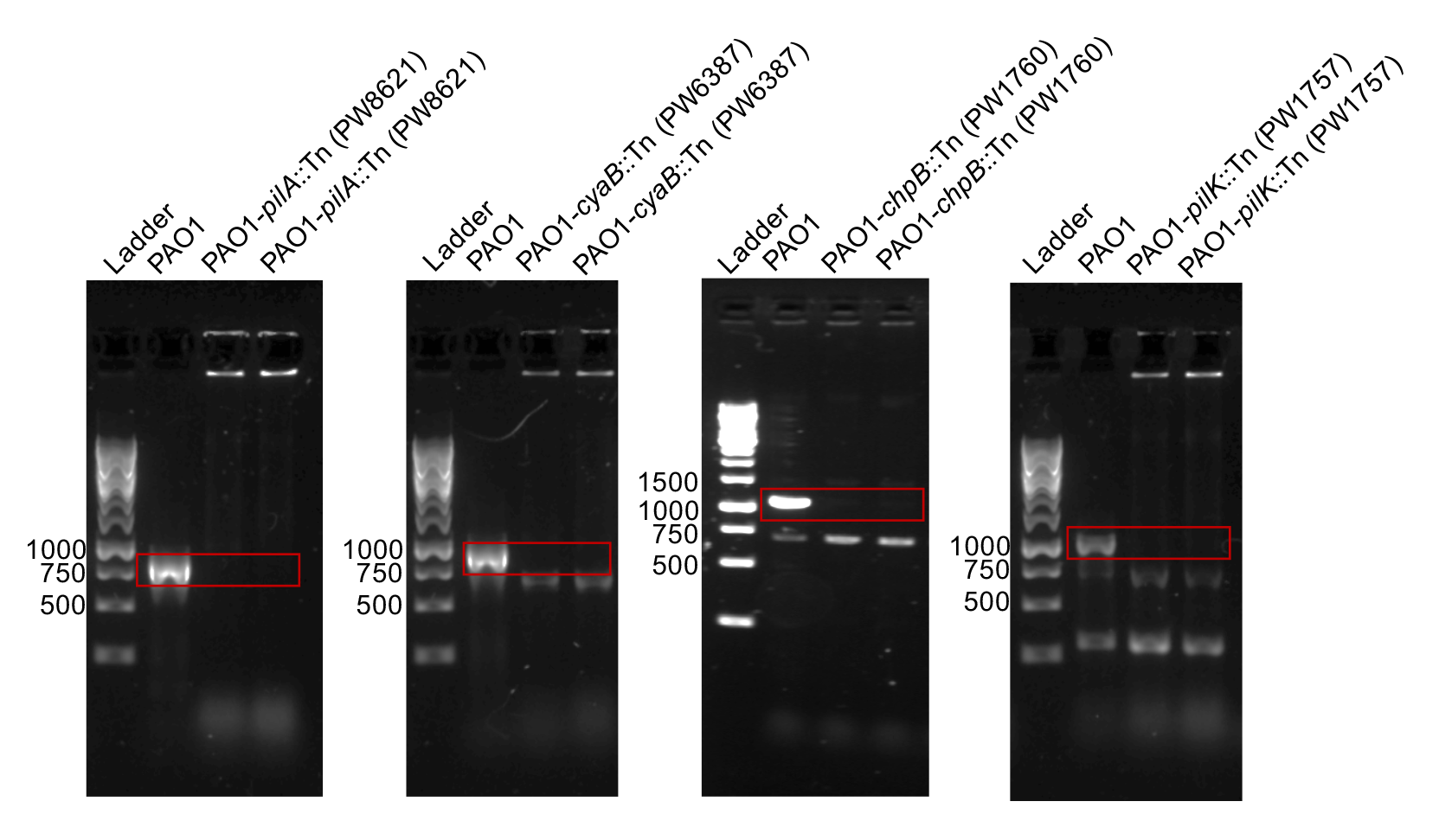

Supplement: S1 Fig — PAO1-pilA::Tn (PW8621), PAO1-cyaB::Tn (PW6387), and PAO1-chpB::Tn (PW1760) were verified by PCR with primers provided by the insertion mutant library database. PAO1-pilK::Tn (PW1757) was verified by PCR with the following primers; pilK flanking primers pilK-F (5'-AGATGCGCAACTCGGTATCC-3') and pilK-R (5'-TTCAGGGTTTCGGCGATCTC-3'). The red square was used to label target products. (TIF) [file ppat.1006392.s006.tif]

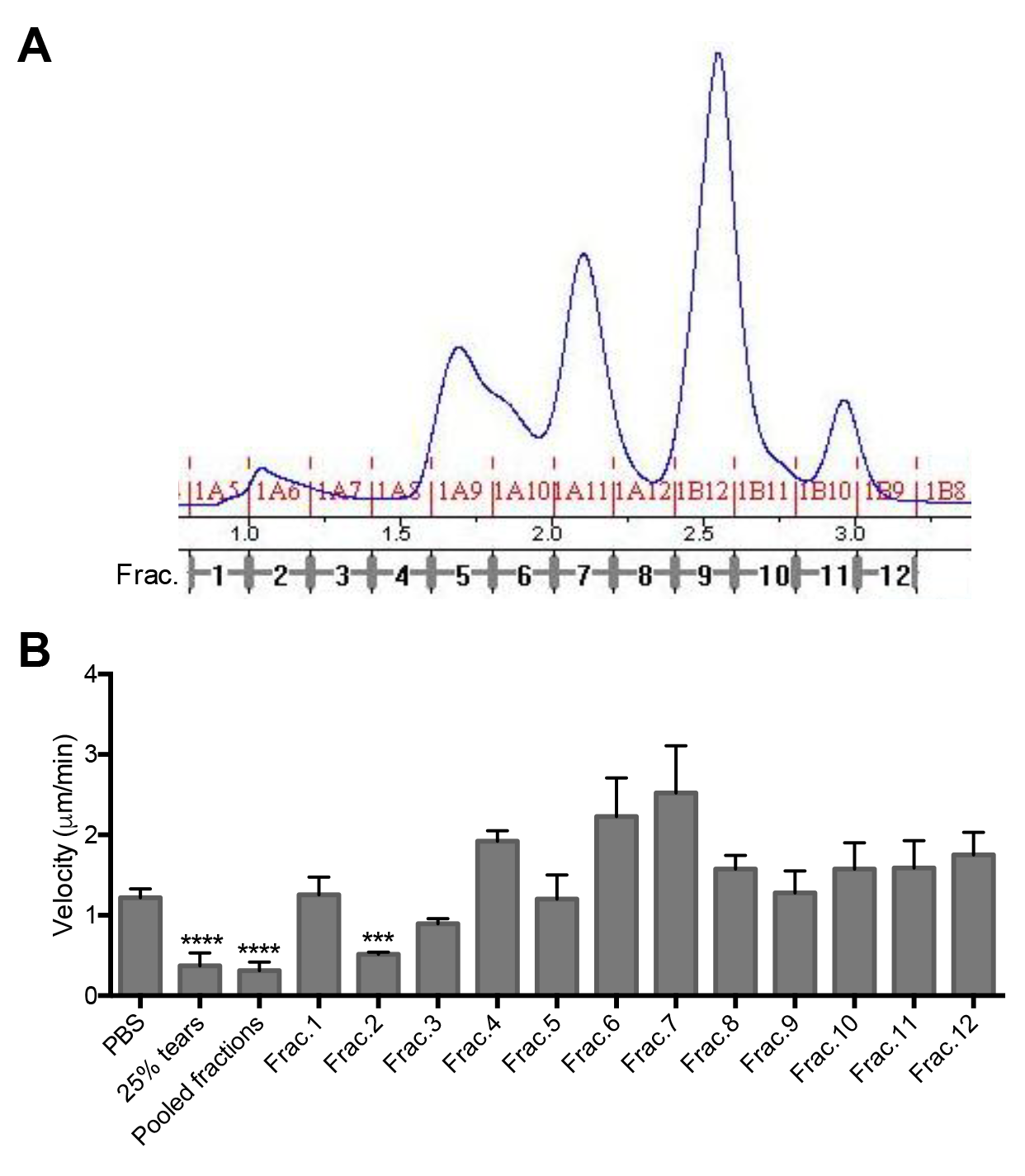

Supplement: S2 Fig — (A) Fractions of human tear fluid separated by size exclusion chromatography (first experiment). (B) Effect of tear fractions on P. aeruginosa PAO1 twitching velocity measured in 5 min videos of each sample. Data are expressed as the mean ± SEM per sample from three independent experiments. Significance was determined using one-way ANOVA with Tukey's post-hoc analysis. **** P < 0.0001, ***P < 0.001. (TIF) [file ppat.1006392.s007.tif]

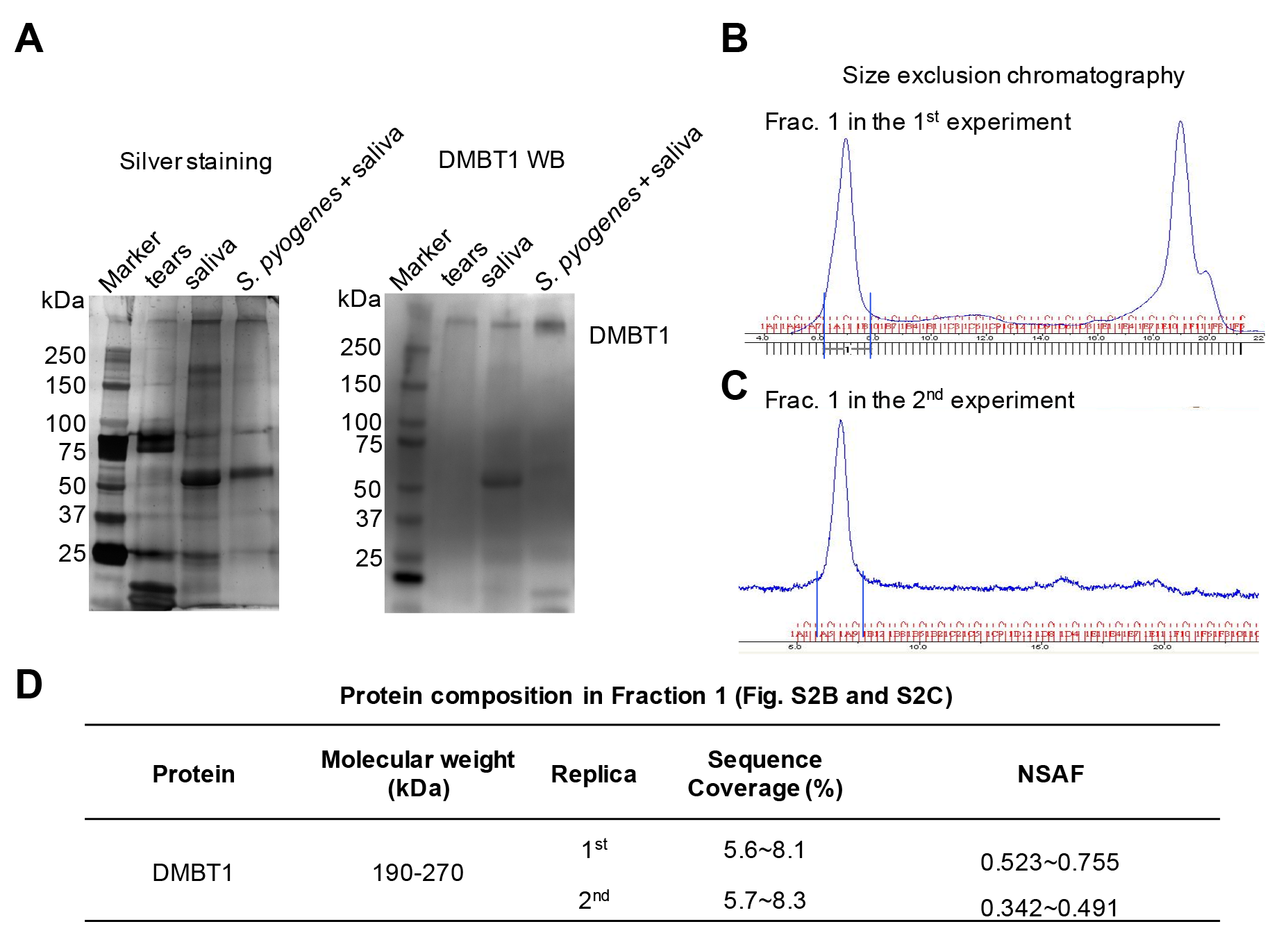

Supplement: S3 Fig — (A) SDS-PAGE with silver stain (left panel) suggested DMBT1 was present after S. pyogenes treatment, and was confirmed by Western immunoblot (right panel) using anti-DMBT1 antibody. (B) and (C) Two independent experiments each showing that size-exclusion chromatography after DMBT1 purification from human saliva using S. pyogenes generated a high Mw fraction (fraction 1). Proteins were separated from aggregated S. pyogenes in human saliva using EDTA (5 mM). (D) Mass spectrometric analysis of fraction 1 after DMBT1 purification from saliva revealed the presence of DMBT1 in two independent experiments. (TIF) [file ppat.1006392.s008.tif]

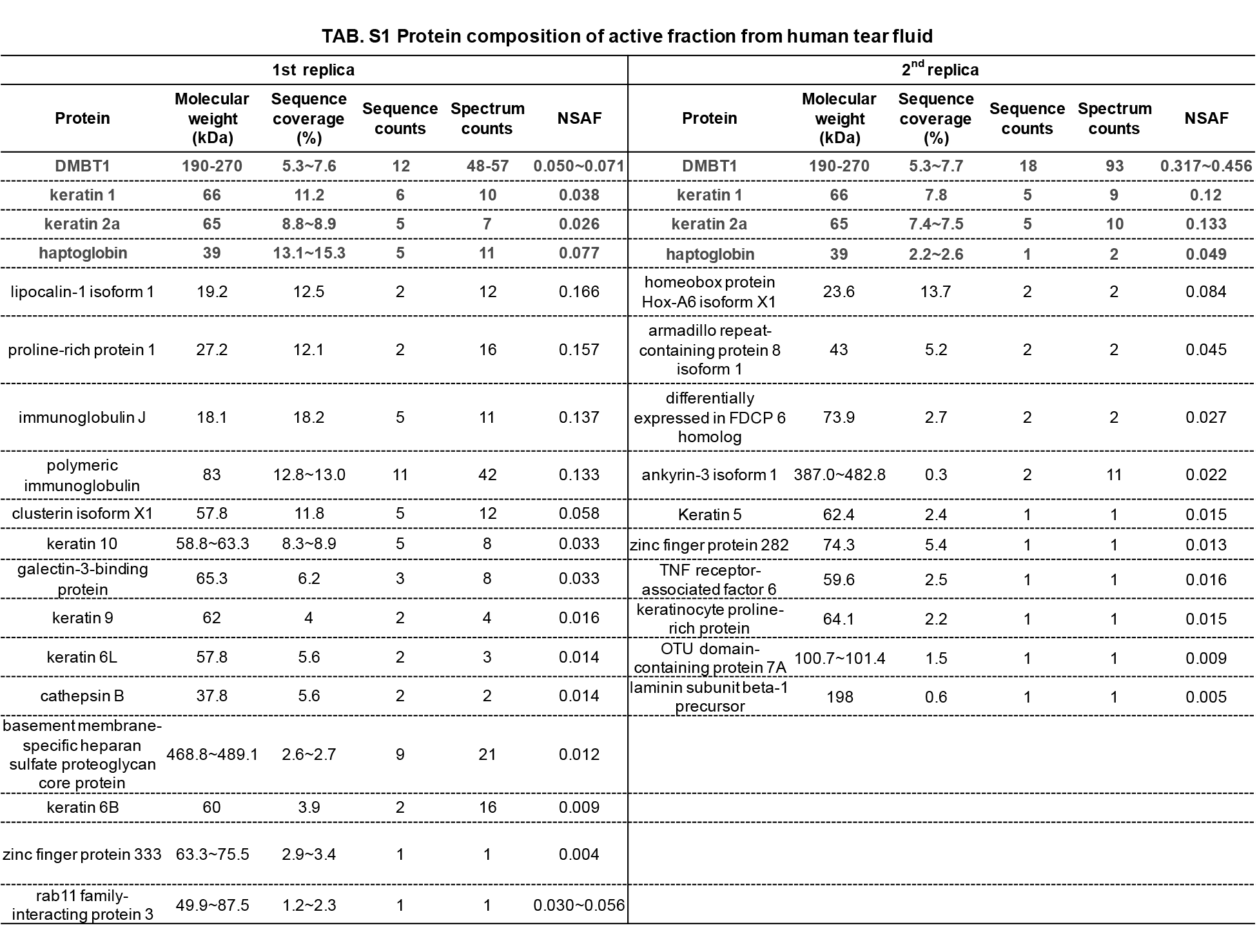

Supplement: S1 Table — Results shown for two independent fractionations of human tear fluid using size-exclusion chromatography. (TIF) [file ppat.1006392.s009.tif]

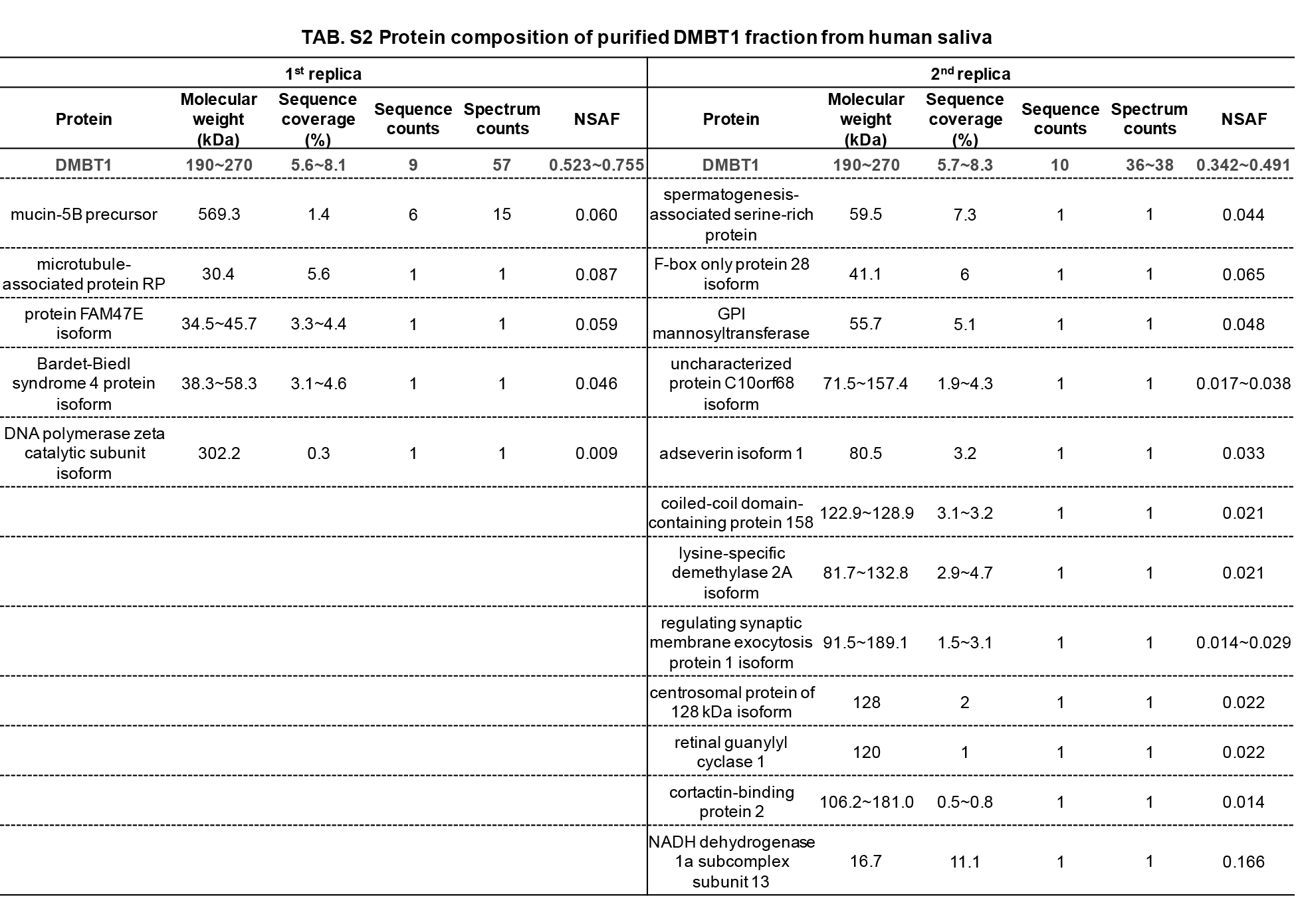

Supplement: S2 Table — Each fraction inhibited twitching motility of P. aeruginosa PAO1. Results represent two independent experiments. (TIF) [file ppat.1006392.s010.tif]
